# A novel signal peptide toolbox for optimized heterologous expression of yeast pheromones in *Bacillus subtilis*

**DOI:** 10.1016/j.jgeb.2025.100551

**Published:** 2025-08-09

**Authors:** Tomislav Vološen, Henri Max Deda, Anica Walther, Philipp F. Popp, Thorsten Mascher, Diana Wolf

**Affiliations:** a General Microbiology, Institute of Microbiology, TUD Dresden University of Technology, Dresden, Germany; b Department of Aquatic Microbiology, Institute of Microbiology, University of Greifswald, Greifswald, Germany; c ScienceOS UG (haftungsbeschränkt), Malschendorfer Str. 4, 01326 Dresden, Germany; d Institute of Biology/Molecular Microbiology, Humboldt-Universität zu Berlin, Berlin, Germany

**Keywords:** Toolbox, Peptide expression, Peptide secretion, Sec system, *Bacillus subtilis*, Yeast peptides

## Abstract

Optimising downstream processes and achieving high product yields are essential for cost-effective and scalable bioproduction, with efficient protein secretion being a critical factor in industrial applications. To address this, we leveraged the widely utilised Sec secretion system of *Bacillus subtilis* to enhance heterologous protein secretion. Given the lack of reliable *in silico* tools for predicting optimal combinations of Sec system signal peptides (SPs) with a protein of interest (POI), we aimed to optimise the secretion efficiency of SP–POI pairings by a toolbox approach. We developed an integrative evaluation vector in which the promoter, SPs, and a coding sequence (CDS) of the POI are easily exchangeable. Further, we generated a toolbox containing 74 SPs naturally present in *B. subtilis* that can easily be integrated into the evaluation vector and thereby fused to the POI. As proof-of-concept, two short peptides from yeast: α-pheromone from *Saccharomyces cerevisiae* and P-pheromone from *Schizosaccharomyces pombe* were chosen as POI and secretion efficiency was measured. Successful expression and secretion of both peptides in *B. subtilis* were verified by an indirect ELISA assay. Eight out of 74 SPs facilitated P-pheromone secretion, while just three effectively enabled the expression of α-pheromone. The maximum observed peptide secretion levels were 43 nM for P-pheromone and 8 nM for α-pheromone. This work demonstrates the necessity of versatile screening approaches to find a matching pairing of the Sec secretion system SP and a POI. We close this gap by providing a robust toolbox with easily exchangeable elements.

## 1 Introduction

The estimated market of technical bulk enzymes produced in industrial biotechnology doubled in the last ten years and was reaching approximately US$11.9 billion in the year 2022.^1^ This economic development increases the need for new technologies and platforms with enhanced speed and cost efficiencies for production. Hereby, extracellular protein secretion is a critical technology for reducing the costs of industrial downstream processing, product recovery, and energy consumption in various industrial applications.^2^ Not surprisingly, optimising protein secretion in industry-relevant organisms is a priority in White Biotechnology processes.^3^

The Gram-positive bacterium *Bacillus subtilis* includes the “generally recognised as safe” (GRAS) status and is widely used as the host for the production and secretion of commercial enzymes.^4^ In 2014, 17 % of total commercial enzymes were produced by *B. subtilis*.^2^ *B. subtilis* harbours four main secretion pathways, of which the Sec secretion pathway is the most prominently used.^5^ Proteins secreted by the Sec pathway carry a secretion-targeting peptide sequence in the N-terminus referred to as signal peptide (SP).^6^ Transmembrane protein translocation through the Sec-pathway in *B. subtilis* is controlled by at least four proteins, including the SecA translocation motor that drives preprotein through the SecY-SecG-SecE channel.^7^ After releasing the C-domain of the SP in the extracytoplasmic space, signal peptidase (SPase) and signal peptide peptidase (SPP) remove the SP from the secreted proteins. After full translocation of the preprotein through the cell membrane, proteins will fold into their native conformation with the help of folding catalysts (e.g., PrsA and SpoIIIJ) and quality control factors (e.g., HtrA-like proteases).^8^.

*B. subtilis* secretes over 173 different proteins through the Sec secretion system. Identifying the optimal SP is a critical factor for the efficient heterologous production and secretion of a protein of interest (POI).^9^, ^10^ Due to the limited understanding of the factors influencing protein secretion and the challenge of identifying a functional pairing with a SP, *in silico* prediction tools for selecting SPs to optimise protein secretion are currently lacking.^11^ To address this, we designed and evaluated a versatile and cost-effectively toolbox to identify the ideal SP for the secretion of any POI in *B. subtilis*. As proof of concept, we chose to produce α- and P-pheromone from *S. cerevisiae* and *S. pombe* in *B. subtilis* to challenge the usability and effectiveness of the toolbox and its included expression evaluation vector. Yeast α- and P-pheromone are 13- and 23-amino acids long peptides, respectively and are functionally essential for mating of haploid yeast cells.^12^, ^13^ Additionally, recombinant secretion of short peptides has not been documented in *B. subtilis* yet*.* We were interested in the optimal secretion of these peptides in order to be able to successfully design an interkingdom cell–cell communication between yeast and *B. subtilis* by using these pheromones as part of a larger research project. To our knowledge, heterologous production of peptides smaller than 25 amino acids and originating from eukaryotes in prokaryotes is novel and can open a wide range of applications in White Biotechnology.

## 2 Materials and methods

### 2.1 Evaluation vector construction

All vectors and oligonucleotides used to create the evaluation vector are listed in Table S1 and Table S2. The evaluation vector was developed from the pBS1C backbone previously published in.^14^ To use this vector as the backbone of the evaluation vector, the *Bsa*I restriction enzyme site was removed with PCR mutagenesis using the oligonucleotides TM3163 and TM3164. Next, xylose inducible promoter P*_xylA_* was amplified with oligonucleotides iG17P051 and iG17P052 from plasmid pBS2E-P*_xylA_* (9A’s),^15^ digested with *EcoR*I-HF and *Bsa*I (*Xba*I 5′ CTAG…3′ overhang), and ligated into the *Bsa*I free variant of pBS1C vector that was previously digested with *EcoR*I-HF and *Xba*I-HF. The resulting vector pBS1C-P*_xylA_* was confirmed by sequencing. pBS1C-P*_xylA_* vector was further developed by integrating *rfp* and *lacZα* genes inside the multiple cloning sites to achieve a three-colour colony screening (red-blue-white) of transformed *E. coli* cells. To do so, a *rfp* gene was synthesised and fused to the IPTG inducible promoter P*_lacI_* (Integrated DNA Technology’s (IDT, Coralville, IA, USA) gBlocks® Gene Fragments DNA synthesis) and inserted into the storage vector pSB1C3 (parts registry: BBa_K823055iGEM) following the BioBrick™ standard. Next, the *rfp* gene was amplified from pSB1C3-[RFP] by using the oligonucleotides iG17P110 and iG17P159 and digested with *Xba*I-HF and *Age*I-HF. The *lacZα* gene was amplified with iG17P055 and iG17P056 from DNA storage vector pSB1C3-Bba_I732902(*lacZα*) and digested with *Age*I-HF and *Pst*I-HF. Genes *rfp* and *lacZα* were cloned into the pBS1C-P*_xylA_* vector, digested with *Bsa*I (*Xba*I 5′ CTAG…3′ overhang) and *Pst*I, resulting in the evaluation vector (pBS1C P*_xylA_*-[P*_lacI_*-RFP-*lacZα*]) that was verified by sequencing. Enzymes and buffers used here to develop the evaluation vector were purchased from New England Biolabs® (NEB, Ipswich, MA, USA).

### 2.2 Construction of the signal peptide toolbox

In this study, a toolbox with SPs from 74 Sec-system transported proteins was established. Oligonucleotides used for SP amplification (Table S3) are designed in BioBrick™ standard and contain a ribosome binding site (RBS) and a linker previously described in.^15^ Amplified SPs were cloned into the storage vector pSB1C3 and transformed with *E. coli* DH10β. Cloning was verified by sequencing. To establish the SP toolbox, all storage vectors containing the SP fused to RBS are isolated from *E. coli*, aliquoted in a concentration of 0.5 ng µL^−1^, and stored at −20 °C. A list of all SPs containing storage vectors is shown in Table S4. To use the toolbox, 4 SP mixes can be prepared. Each mix contains 20 different SPs. Every SP subset/mix was amplified by using the oligonucleotides TM4487 and iG17P039 in an adjusted multi-template PCR reaction shown in Table S5. Next, amplified SP mix was digested with *Xba*I and *Age*I, cloned into the evaluation vector carrying the POI, and transformed with *E. coli*. Fifty white *E. coli* colonies per SP subset were picked for overnight cultures and plasmid isolation. Isolated plasmids were transformed with *B. subtilis*. Again, fifty colonies of each SP subset were picked, and a starch assay was performed to check the correct integration of constructed plasmids into the *amyE* locus of the *B. subtilis* genome. In the next step, screening for SPs that are responsible for POI secretion was performed by indirect ELISA assay. SPs of producing cells were identified by sequencing the PCR products of the SP region.

### 2.3 Growth conditions

All strains used in this study are listed in Table 1. *B. subtilis* was grown at 37 °C with aeration in Lysogeny broth LB broth (Luria/Miller, Carl Roth) [tryptone 10 g L^-1^, yeast extract 5 g L^-1^, NaCl 10 g L^-1^] or MNGE media [88.2 % 1 × MN media (1.36 % (w/v) dipotassium phosphate x 3 H_2_O, 0.6 % (w/v) monopotassium phosphate, 0.1 % (w/v) sodium citrate·H_2_O)), 1.9 % glucose, 0.19 % potassium glutamate, 0.001 % (w/v) ammonium ferric citrate, 0.005 % (w/v) tryptophan, and 0.035 % (w/v) magnesium sulphate] or Edinburgh Minimal Medium (EMM) containing 3.75 g L^-1^ glutamic acid as the nitrogen source.^16^ *E. coli* was grown at 37 °C with aeration in Lysogeny broth (LB broth). Solid media contained 1.5 % (w/v) agar. Selection media for *E. coli* contained ampicillin (100 µg mL^−1^). To have a blue-red-white screening of successful *E.* coli transformation, agar plates contained 1 mM IPTG, 100 µg mL^−1^ X-Gal, and 100 µg mL^−1^ ampicillin. Selection media for *B. subtilis* contained chloramphenicol (5 µg mL^−1^) or kanamycin (10 µg mL^−1^). Genetic modification of *E. coli* and *B. subtilis* was performed as described previously.^17^, ^18^ Colonies were transferred to starch agar plates to show successful integration of the pBS1C constructs into the *amyE* locus of the *B. subtilis* genome by starch assay with Lugol’s solution (Sigma Aldrich, Germany).

**Table 1:** *B. subtilis* strains used in this study.

Strain	Description	Source/Reference
*B. subtilis* W168	WT, *trpC2*	Laboratory stock
*B. subtilis* WB800N	*nprE aprE epr bpr mpr*::*ble nprB*::*bsr* Δ*vpr wprA*::h*yg* (Neo^R^)	^19^
TME4009	*E. coli* DH10β pUC57-α-pheromone_H_	This study
TME4010	*E. coli* DH10β pUC57-α-pheromone_MF_	This study
TME4011	*E. coli* DH10β pUC57-α-pheromone_R_	This study
TME4012	*E. coli* DH10β pUC57-P-pheromone_R_	This study
TME4013	*E. coli* DH10β pUC57-P-pheromone_H_	This study
TME4014	*E. coli* DH10β pUC57-P-pheromone_MF_	This study
TMB6298	W168 *amyE*::pBS1C-P*_liaI_*-*fliZ*-P-pheromone_MF_	This study
TMB6301	W168 *amyE*::pBS1C-P*_liaI_*-*ybxI*-P-pheromone_MF_	This study
TMB6315	W168 *amyE*::pBS1C-P*_liaI_*-*fliZ*-P-pheromone_H_	This study
TMB6318	W168 *amyE*::pBS1C-P*_liaI_*-*ybxI*-P-pheromone_H_	This study
TMB6423	WB800N *amyE*::pBS1C-P*_liaI_*-*yhcR*- P −pheromone_MF_	This study
TMB6424	WB800N *amyE*::pBS1C-P*_liaI_*-*ybxI*-P-pheromone_MF_	This study
TMB6425	WB800N *amyE*::pBS1C-P*_liaI_*-*fliZ*-P-pheromone_H_	This study
TMB6426	WB800N *amyE*::pBS1C-P*_liaI_*-*ybxI*-P-pheromone_H_	This study
TMB6427	WB800N *amyE*::pBS1C-P*_liaI_*-*yhcR*-α-pheromone_H_	This study
TMB6428	WB800N *amyE*::pBS1C-P*_liaI_*-*yfkD*-α-pheromone_H_	This study
TMB6429	WB800N *amyE*:: pBS1C-P*_liaI_*-*yfkN*- α-pheromone_H_	This study
TMB6430	WB800N *amyE*::pBS1C-P*_liaI_*-*yhcR*- α-pheromone_MF_	This study
TMB6431	WB800N *amyE*::pBS1C-P*_liaI_*-*yfkD*- α-pheromone_MF_	This study
TMB6432	WB800N *amyE*::pBS1C-P*_liaI_*-*yfkN*- α-pheromone_MF_	This study

MF-most frequent codon usage frequency; H-harmonised codon usage frequency.

### 2.4 Codon adaptation

Codon sequences of α- and P-pheromones from *S. cerevisiae* and *S. pombe* were adapted for expression in *B. subtilis*. Codon optimisation was performed by comparing codon usage frequencies between *B. subtilis* and the yeasts *S. cerevisiae* and *S. pombe*. Codon usage frequencies were obtained from Codon Usage Databases.^20^ Three variations of codon-optimised sequences for α- and P-pheromones were designed. The first sequence contains the most frequent codon sequence that has the highest codon usage frequency in *B. subtilis,* which indicates the highest speed of translation in *B. subtilis*. The robust codon sequence has the lowest possible codon usage frequency, indicating the slowest speed of translation. Furthermore, a harmonised coding sequence was designed by comparing and matching the codon usage frequency of the *S. cerevisiae* and *S. pombe* to the codon usage frequency in *B. subtilis,* resulting in a translation speed similar to both yeast and bacteria. α- and P-pheromone protein sequence, codon sequence, and optimised codon sequences are shown in Fig. S1 and Fig. S2. Designed codon sequences were artificially synthesised by BioCat (Heidelberg, Germany) and donated in the pUC57 vector for storage.

### 2.5 Construction of *B. subtilis* α- and P-pheromone secretion strains

*B. subtilis* was genetically modified to secrete the α- and P-pheromones by using the evaluation vector and the SP toolbox. Strain construction occurred in three steps. First, restriction cloning replaced the *lacZ* gene in the evaluation vector with α- or P-pheromone coding sequences. To do so, the coding sequence and evaluation vector were digested with *Pst*I and *Ngo*MIV, ligated and transformed with *E. coli*. Blue-red screening of clones and sequencing of plasmid constructs confirmed the successful cloning. Second, we determined the SPs that potentially secrete α- and P-pheromone by using the SP toolbox developed in this study. Finally, we exchanged the promoter in the plasmids, including the optimal SP from P*_xylA_* to the P*_liaI,_* to ensure antibiotic inducible expression of the pheromones and circumvent leakiness of P*_xylA_*. The evaluation vector carrying P*_xylA_*, RFP gene, and α- or P-pheromone was digested with *Eco*RI-HF and *Ngo*MIV-HF. The desired SP was digested with *Xba*I*-HF* and *Age*I-HF. At the same time, the promoter P*_liaI_* was amplified with oligonucleotides TM2895 and TM2896 (Table S2) and digested with *Eco*RI-HF and *Spe*I-HF. Constructs were confirmed by sequencing and transformed with *B. subtilis* W168 (laboratory standard strain) and WB800N strain (an eight-protease-deficient strain).^19^

### 2.6 Heterologous expression of α- and P-pheromones in *B. subtilis*

Established α- and P-pheromone-producing *B. subtilis* strains were used to perform expression studies. Overnight cultures, including appropriate antibiotic chloramphenicol (5 µg mL^−1^) for selection, were diluted 1:100 in fresh LB or EMM media.^21^ 200 µL of the culture dilution was aliquoted in wells of a transparent 96-well microplate (Greiner Bio-One, Kremsmünster, Austria) and cultivated at 37 °C for 3 h with agitation. Then, peptide expression was induced by adding xylose in a final concentration of 0.5 % (w/v) or 100 µg mL^−1^ of bacitracin zinc salt (Sigma), and cultivation with agitation was continued for 3 h. Expression was stopped by adding 200 nM Tris-HCl pH 8.0 buffer, 10 mM EGTA pH 8.0, and cOmplete Protease inhibitor cocktail (Merck) or Halt™ Protease-Inhibitor Cocktail (Thermo Fischer Scientific). Finally, the cultivation was inactivated by heating the 96-well plate to 80 °C for 3 min, and cells were removed by centrifugation at 1800 × g for 15 min. 100 µL of the remaining supernatant was used for an indirect ELISA assay to check and quantify the pheromone secretion.

### 2.7 Indirect ELISA assay

Visualisation and quantification of α- and P-pheromone concentration were conducted by a modified indirect ELISA protocol adopted from.^22^ In brief, 100 µL of culture supernatant was transferred to each well of an ELISA plate (High Binding 96 Chimney Well Bottom Plate, Greiner Bio-One, Kremsmünster, Austria). Additionally, 100 µL of synthetic α- or P-pheromones (Peptides & Elephants GmbH, Berlin, Germany and David’s Biotechnology GmbH, Regensburg, Germany were added in a concentration range from 2.47 nM to 200 nM. After the generation of a four-parameter-dose–response calibration curve, synthetic peptides were added in a range from 0.21 nM to 50 nM. As a positive control, the supernatant of the *B. subtilis* wild type strain was supplemented with synthetic α- and P–pheromone and added to the ELISA plate. Next, the plate was covered with a lid and stored at 4 °C overnight or at room temperature for 2 h. After 2 h, wells were washed three times with 200 µL of PBST buffer (8 g NaCl, 0.2 g KCl, 1.44 g Na_2_HPO_4_, 0.24 g KH_2_PO_4_, 0.1 ml Tween20, pH 7.2) and residual binding sites on ELISA plates were blocked with 100 µL of 1 % (w/v) BSA (bovine serum albumin) dissolved in PBST buffer for 2 h at room temperature. The ELISA plate was rewashed three times with PBST buffer, and 100 µL of 0.4 µg mL^−1^ α- or P-pheromone antibody from rabbit (Peninsula Laboratories International, Inc. (currently BMA Biomedicals, Switzerland) and David’s Biotechnology) dissolved in 0.5 % (w/v) BSA in PBST was added. After 1 h, wells were rewashed and 100 µL of Anti-Rabbit IgG (H + L) HRP Conjugate (working solution 1:2500 diluted in 0.5 % BSA in PBST buffer) (Promega) was added. After 1 h, the last washing step was performed and the peroxidase reaction was started by adding 100 µL of Pierce™ TMB substrate kit solution in each well. The reaction was stopped by adding 100 µL of 2 M sulfuric acid. Subsequently, absorbance was measured at 450 nM in a Synergy Neo3 Hybrid Multimode Microplate Reader from BioTek (Winooski, VT, USA).

## 3 Results

### 3.1 Establishment of the evaluation vector and signal peptide toolbox

**Fig. 1:**
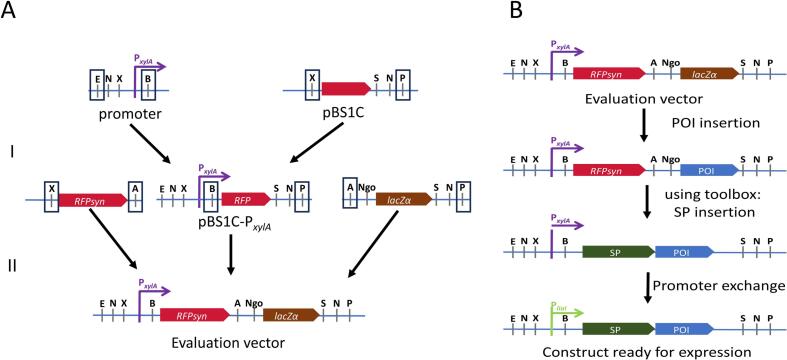
Evaluation vector establishment and application. A Schematic overview of the multiple cloning site development into the evaluation vector. (I) Xylose inducible promoter was fused into the BsaI-free pBS1C background vector. (II) Multiple cloning site was enriched with synthesized (fused to P_lacI_) rfp_syn_ and lacZα to establish the evaluation vector. Black box indicate which restriction enzyme was used for cloning. B Schematic overview of evaluation vector application. POI-protein of interest, SP-signal peptide, E-EcoRI, N-NotI, X-XbaI, B-BsaI (XbaI overhang), A-AgeI, Ngo-NgoMIV, S-SpeI and P-PstIA.

To develop a highly versatile and easy-to-use vector for heterologous protein expression in *B. subtilis*, we utilised the well-established pBS1C.^14^ Desired characteristics of this vector are a moderate size (<7 kb), a copy number of 20–30 in *E. coli*, and the possibility of integration into the *amyE* locus of *B. subtilis,* for which the starch assay is a low-cost screening method to determine *B. subtilis* mutants. First, we inserted the well-characterised xylose inducible promoter, P*_xylA,_* in the adopted BioBrick^TM^ RFC10 standard into pBS1C to enable the exchangeability of the promoter if desired (Fig. 1A). In the next step, we placed gene sequences of *rfp* and *lacZα* into the vector to establish the final evaluation vector pBS1C-P*_xylA_*-*rfp*-*lacZα,* in which the SP and the sequence of POI can be cloned via the restriction sites *Age*I and *Ngo*MIV (Fig. 1A and Fig. S3).

Based on the *rfp* and *lacZα* genes in the MCS, the evaluation vector provides a quick identification of integrated SP and POI inserts via red-blue colony screening in *E. coli* (Fig. 1B). When desired, the inducible P*_xylA_* promoter that drives the POI expression in the evaluation vector can be exchanged easily due to the presence of the *Bsa*I restriction enzyme cleavage site (Fig. 1B). For this study, we first introduced α- and P-pheromone gene sequences from *S. cerevisiae* and *S. pombe* as POI into the evaluation vector. Codon adapted sequences of both pheromones were utilised to ensure efficient heterologous expression in *B. subtilis*. Thus, we designed a set of coding sequences adapted for heterologous expression in *B. subtilis* (Fig. S1 and S2). After coding sequences were inserted into the evaluation vector, the SP toolbox was used to randomly introduce SP sequences in front of α- and P-pheromone encoding sequences, respectively. In general, the SP toolbox is not a predictive tool designed to directly estimate SP-POI relationships. However, to address the lack of computational SP prediction tools, we developed an experimental approach that is both fast and easily implementable, enabling the efficient identification of the optimal SP-POI pairing. 74 SP sequences, divided into four SP-mix libraries, were generated (Table 2). Each library contains 0.5 ng µl^−1^ DNA concentration of each SP pre-cloned in pSB1C plasmids (Table 2).

**Table 2:** Sec secretion signal peptides used in this study are divided alphabetically in signal peptide mix subsets.

Signal peptide mix: Subset 1									
AmyE	AspB	BglS	Bpr	CccA	CitH	Csn	DacB	DacF	DltD
Epr	FliL	FliZ	GlpQ	LipA	LytB	LytC	LytD		
Signal peptide mix: Subset 2									
LytR	Mdr	Mpr	NprE	PbpX	Pel	PelB	PenP	PhoA	PhoB
PhrA	PhrC	PhrF	PhrG	PhrK	RpmG				
Signal peptide mix: Subset 3									
SacB	SacC	SleB	SpollD	SpollP	SpollQ	SpollR	TyrA	Vpr	WapA
YbbC	YbbC	YbbR	YbdG	YbdN	YbfO	YbxI	YdbK	YdhT	YdjM
Signal peptide mix: Subset 4									
YdjN	YfhK	YfjS	YfkD	YfkN	YhcR	YhdC	YhfM	YhjA	YjcN
YjdB	YjfA	YjiA	YkoJ	YkvV	YkwD	YlaE	YlbL	YlqB	YlxF

SP names derive from the protein names for which the SPs designate secretion in their native context. Additionally, each SP sequence carries an RBS, a spacer sequence optimised for expression in *B. subtilis,* followed by the start codon. Protein sequences of all 74 signal peptides can be found in Table S5. Furthermore, each SP sequence holds identical flanking DNA sequences up- and downstream that allow multi-template PCR to enrich the SP mix subsets while using the toolbox.

### 3.2 Evaluation of the secretion peptide toolbox

**Fig. 2:**
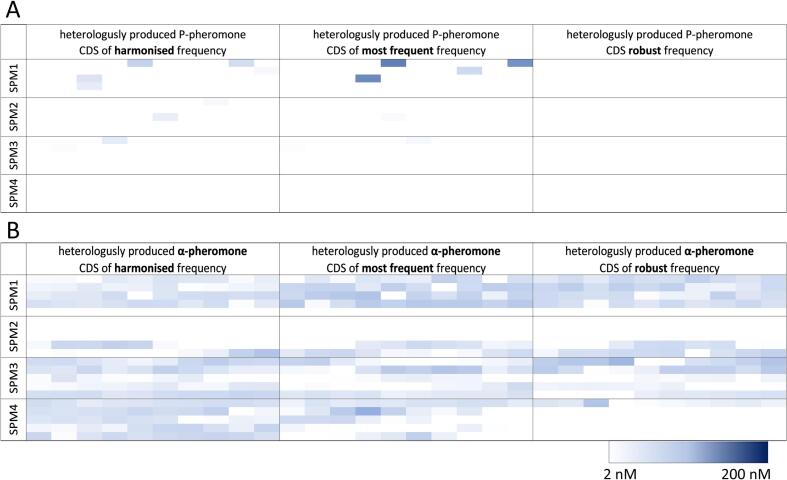
P- and α-pheromone secretion screening by ELISA assay. ELISA assay was performed on 1200 colonies to screen the SP-Mix subsets (SPM1-4), each fused to the three optimised coding sequences (CDS), for (A) P-pheromone and (B) α-pheromone secretion in *B. subtilis*. Cultures were grown in LB media and expression/secretion was induced with 0.5 % (v/v) xylose. Heat map represents the average value of biological duplicates. Colour gradient is in the range from a minimum pheromone concentration of 2.47 nM to a maximum concentration of 200 nM.

Enriched peptide mix subsets were digested with *Xba*I and *Age*I, cloned into the evaluation vector already carrying the yeast pheromones, and transformed with *E. coli*. Afterwards, white colonies, potentially carrying correct SP-POI constructs, were picked for plasmid isolation. Plasmids were transformed to *B. subtilis* and 50 colonies for each SP subset were transferred to starch agar plates to check the successful plasmid integration into the *B. subtilis* genome with Lugoĺs solution. *B. subtilis* colonies, including the artificial SP-POI construct within the genome, were then transferred to 5 µg mL^−1^ chloramphenicol agar plates for storage and subsequent expression/secretion experiments. To evaluate expression of SP-POI constructs, generated *B. subtilis* strains were grown to the exponential phase and induced with the addition of 0.5 % (w/v) xylose. Supernatants were collected after 3 h and used for indirect ELISA assays to probe successful secretion of α- and P-pheromones (Fig. 2). Successful expression and secretion of P-pheromone was conducted by using SP-mix subsets 1, 2, 3, and 4 in combination with the harmonised and most frequent coding sequence of P-pheromone (Fig. 2A). Among SP mixtures, the majority of secreting P-pheromone clones belong to SP-mix subset 1. On the other hand, no secretion of P-pheromone for SP-mix subset 4 was observed (Fig. 2A). Further, only 14 clones out of 600 tested were able to secrete any P-pheromone, highlighting that the optimal SP-POI combination is critical for secretion of heterologously expressed proteins in *B. subtilis* (Fig. 2 and Table 3). However, the pheromone coding sequence and therein embedded codon usage of *B. subtilis* play an important role as well in the expression and secretion of artificial proteins. The darker colour in the heat map indicates a higher concentration of P-pheromone encoded by the most frequent adapted coding sequence compared to the harmonised coding sequence. For the robust coding sequence, no secreted P-pheromone could be detected (Fig. 2A). To identify the SPs yielding in P-pheromone secretion, the 14 clones were identified by sequencing. In the case of α-pheromone secretion, we detected the secreted peptide for every optimised coding sequence across each SP-mix subset (Fig. 2B). However, all determined yields for α-pheromone production resulted in low concentrations at the limit of detection (Fig. 2B). Since none of the tested colonies showed a high concentration of secreted α-pheromone, random clones were chosen for sequencing and thereby the identification of SP responsible for peptide secretion (Table 3). In total, seven SPs that contribute to the secretion of P-pheromone and three SPs responsible for α-pheromone secretion in *B. subtilis* were identified (Table 3). In summary, the toolbox developed in this study provides a powerful tool to screen a versatile variety of SPs in a single step and subsequently identify promising pairs of SP(s) and POI for successful secretion.

**Table 3:** Identified signal peptides responsible for α- and P-pheromone peptide secretion.

SP for P-pheromone secretion	SP for α-pheromone secretion
BglS	YfkD
Bpr	YfkN
DacF	YhcR
FliZ	
SacC	
YbxI	
YdjN	

The signal peptide protein sequence is shown in Table S5.

### 3.3 Enhancement of pheromone production by fine-tuning expression parameters

**Fig. 3:**
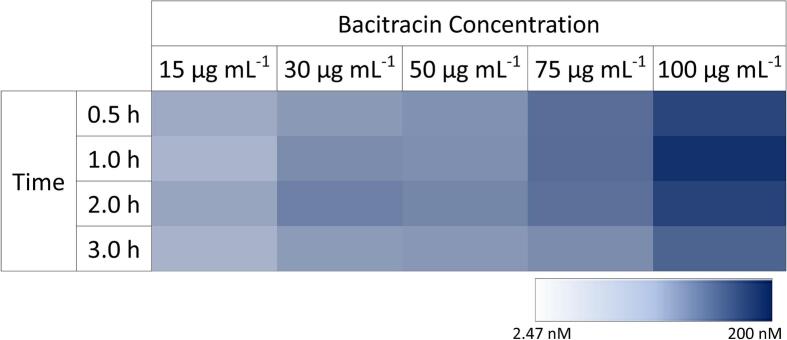
Characterisation of bacitracin-induced P-pheromone production in B. subtilis. Strain B. subtilis W168 P_liaI_-ybxI-P_H_ was induced with bacitracin (concentration range 15–100 µg mL^−1^) and samples were collected after 0.5, 1.0, 2.0, 3.0 h and analysed by indirect ELISA assay. Heat map represents the average value of triplicates. Colour gradient is in the range from minimum P-pheromone concentration 2.47 nM to maximum concentration of 200 nM P-pheromone.

**Fig. 4:**
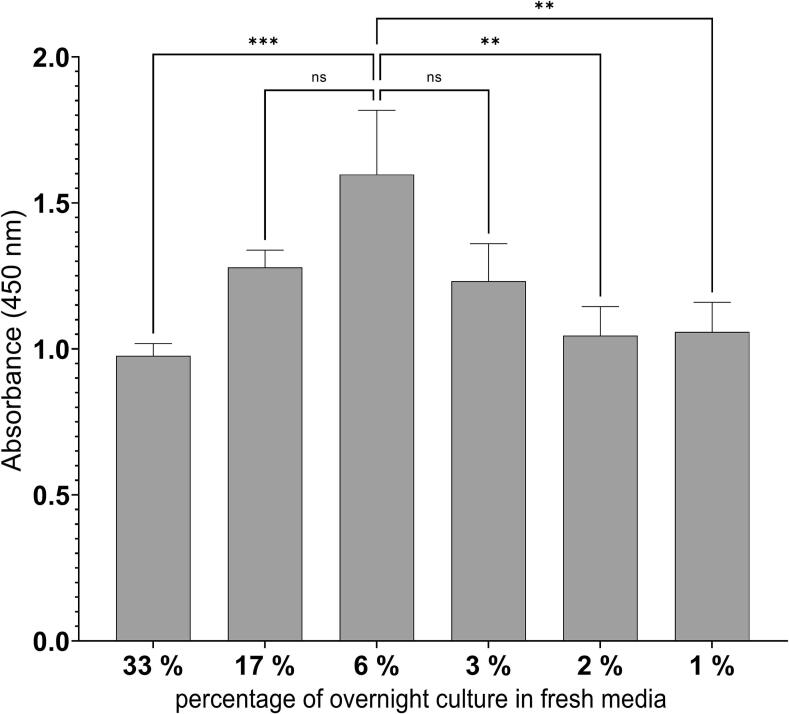
Influence of the *B. subtilis* culture start optical density on the P-pheromone production. Overnight culture of B. subtilis W168 P*_liaI_*-*ybxI*-P_H_ was mixed with fresh media in different ratios (shown as the percentage of overnight culture in fresh media) and expression started after 3 h of cultivation by induction with bacitracin (100 µg mL-1). Samples were harvested 1 h after the induction and analyzed with indirect ELISA. Experiments were performed as technical triplicates. Statistical difference was determined using one-way ANOVA followed by Bonferroni’s multiple comparisons test to do pairwise comparison between start optical densities. Error bars present the standard deviation from the mean value. ns = not significant, ** = p < 0.01, and *** = p < 0.001.

The next step in secretion improvement consisted of the heterologous pheromone expression under the control of an antibiotic-regulated promoter. Here, we chose the well-studied antibiotic inducible promoter P*_liaI_* of *B. subtilis* to increase the secretion of α- and P-pheromone. The P*_liaI_* promoter has been selected due to the high induction activity (∼100-fold) after adding the antibiotic bacitracin.^23^, ^24^ Reasoned by the low secretion of α–pheromone, we decided to optimise and test the expression/secretion level of P-pheromone only by using P*_liaI_*. The optimised (harmonised and most frequent) coding sequence of P-pheromone fused to the seven selected SPs (Table 3) were fused to P*_liaI_*. Then, generated strains were grown and induced with the antibiotic bacitracin with concentrations ranging from 15 to 100 µg mL^−1^ to start heterologous expression and secretion of P-pheromone in *B. subtilis*. We found that expression of the construct P*_liaI_*-*ybxI*-P_H_ resulted in the highest concentration of secreted P-pheromone (Fig. S4). Additionally, the next best-performing expression cassette is P*_liaI_*-*ybxI*-P_MF,_ indicating optimal SP for P-pheromone secretion is YbxI, which will be used for further experiments in this study (Fig. S4). After the identification of YbxI-SP as the best-performing SP for P-pheromone secretion, we started to characterise the concentration of bacitracin that works best for optimal promoter induction and peptide expression time interval (Fig. 3). In general, increasing bacitracin concentrations resulted in increasing P-pheromone secretion in a time range between 0.5 and 3 h. The highest amount of secreted P-pheromone was detected after the addition of 100 µg mL^−1^ of bacitracin and an expression time of 1 h (Fig. 3). A longer period (> 3h) of expression cultivation resulted in a decrease of P-pheromone secretion, which can be observed for all tested bacitracin concentrations. The next question we asked ourselves was to what extent the optical density of the cells to be induced affects P-pheromone secretion (Fig. 4). To do so, different amounts of *B. subtilis* overnight culture (33 %, 17 %, 6 %, 3 %, 2 % and 1 %) were diluted in fresh media and cultivated for 3 h before 100 µg mL^−1^ bacitracin was added. One hour after induction, supernatants were collected and analysed by ELISA. We found that an amount of 3 % to 17 % of overnight cells, diluted into fresh media and induced with bacitracin, resulted in the highest levels of P-pheromone secretion (Fig. 4). The most substantial presence of P-pheromone in culture supernatant was detected when 6 % of cells were used to inoculate the fresh media.

**Fig. 5:**
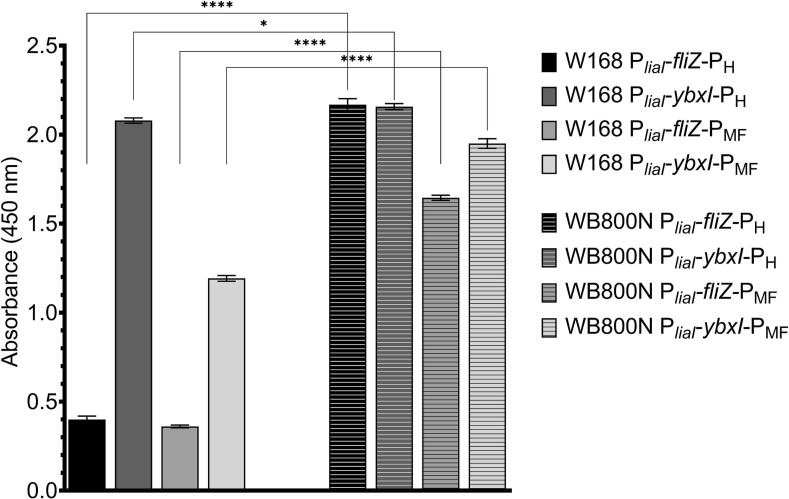
Difference in secretion performance between *B. subtilis* strains W168 and WB800N. Production of P-pheromone with best performing signal peptides with either one of two coding sequences (H-harmonised, MF-Most frequent) was analysed in *B. subtilis* strains WB800N and W168. Indirect ELISA was used to detect the secreted P-pheromone. Experiments were performed as technical triplicates. Statistical difference was determined using an unpaired *t*-test with Welch's correction test to do comparison between two *B. subtilis* species. Error bars present standard deviation from the mean value, * = *p* < 0.05, and **** = *p* < 0.0001.

In line with our observation of decreasing P-pheromone concentration in supernatants after an expression time of more than 1 h and 75 or 100 µg ml^−1^ of bacitracin (see Fig. 3), we thought about the possibility of peptide instability or degradation by extracellular proteases of *B. subtilis* W168. To investigate if the extracellular proteases are a reason for lower P-pheromone amounts in supernatants, we transferred the most promising plasmids containing P-pheromone secretion constructs to the strain *B. subtilis* WB800N that is deficient for eight extracellular proteases.^25^ Heterologous expression and secretion of P-pheromone in *B. subtilis* WB800N was compared to the secretion conducted in *B. subtilis* W168 (Fig. 5). We found a significantly higher amount of P-pheromone in supernatants of *B. subtilis* WB800N strains compared to *B. subtilis* W168 strains. These results indicated that, even though we added a protease inhibitor cocktail directly after harvesting the supernatants of *B. subtilis* W168, protease activity was high enough to reduce the pheromone concentration over time of cultivation. However, wild-type and WB800N strains including P*_liaI_*-*ybxI*-P_H_ show similar secretion levels (Fig. 5). This result is due to the saturation limit (outside of the linear working range) of the plate reader and the necessary optimisation of the indirect ELISA assay. Nevertheless, these results again indicate that expression and secretion of P-pheromone under P*_liaI_* and SP YbxI seem to be the best. Additionally, *B. subtilis* WB800N was the strain of choice for further pheromone secretion experiments in this work.

**Fig. 6:**
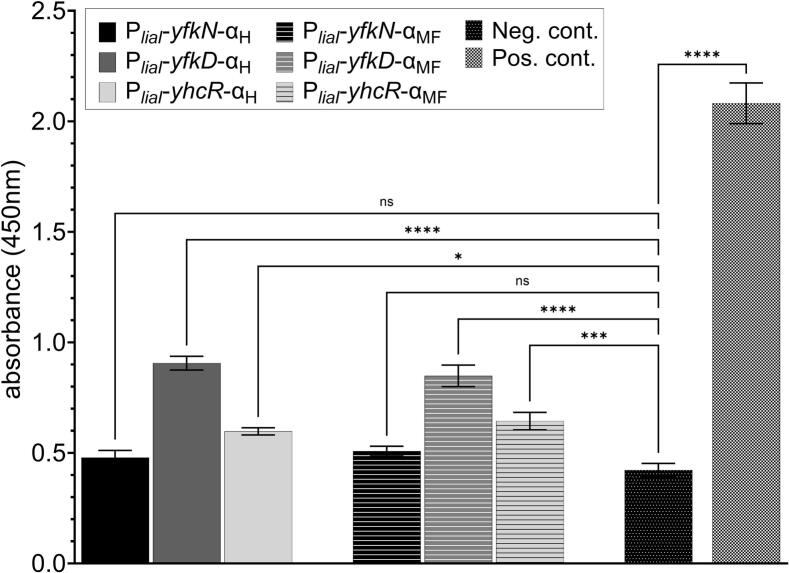
α-pheromone secretion in *B. subtilis* WB800N. α-pheromone detected in supernatant of *B. subtilis* WB800N strains with indirect ELISA. Negative control (Neg. cont.) is supernatant of *B. subtilis* WB800N non-producer strain. Strain is missing the expression/secretion cassette. Positive control (Pos. cont.) is the standard concentration (100 nM) of synthetic α −pheromone added into the supernatant of *B. subtilis* WB800N non-producer strain. Experiments were performed as technical triplicates. Statistical difference was determined using one-way ANOVA followed by Bonferroni’s multiple comparisons test to do pairwise comparison between negative control and α-pheromone producing strains. Error bars present standard deviation from the mean value, ns = not significant, * = *p* < 0.05, *** = *p* < 0.001, and **** = *p* < 0.0001.

As next step, the optimisation and characterisation results of the strain *B. subtilis* WB800N, including the bacitracin-inducible promoter P*_liaI_* fused to the SP P-pheromone (harmonised or most frequent sequence), were transferred to improve the expression and secretion of α-pheromone. Thus, the SP-α–pheromone secreting cassette was fused with P*_liaI_* and transformed with *B. subtilis* WB800N. Again, secretion studies were performed by indirect ELISA and the results of the α-pheromone secretion are shown in Fig. 6. In general, α–pheromone was successfully secreted by the used strains (see Fig. 6). However, the amount of secreted α–pheromone is low compared to the negative control for which supernatant of *B. subtilis* WB800N non-producer strain was used. The highest amount of α–pheromone was achieved with YfkD SP. Furthermore, in contrast to the P-pheromone secretion strains, there is no dominating α-pheromone coding sequence and similar amounts of α–pheromone were observed for both the most frequent (MF) and harmonised (H) sequence. It was decided to use the harmonised coding sequence of α-pheromone for further experiments.

**Fig. 7:**
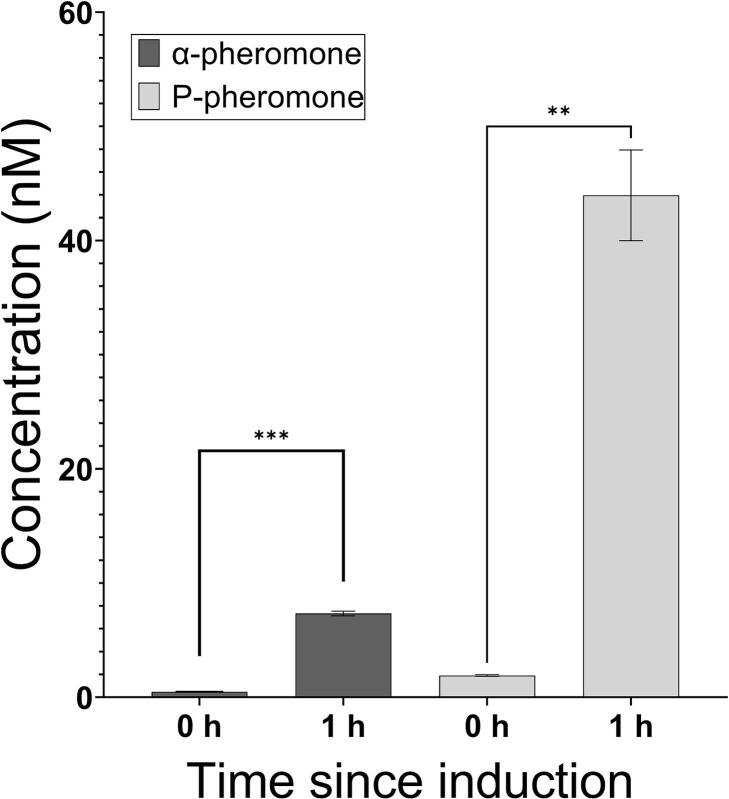
Concentration of secreted α- and P-pheromone at various time points. Detected concentration of heterologously secreted α- and P- pheromone in *B. subtilis* WB800N P*_lia_*_I_-*ybxI*-P_H_ for P-pheromone and P*_liaI_*-*yfkD*-α_H_ for α-pheromone. Indirect ELISA was used to determine the α- and P-pheromone concentrations. Experiments were performed as technical triplicates. Error bars present the standard deviation from the mean value. Statistical significance was determined with an unpaired *t*-test with Welch’s correction (** = p < 0.01, *** = p < 0.001).

Finally, to determine the precise concentration of secreted α- and P-pheromone in supernatants, a four-parameter-dose–response curve was established, which maps a concentration range from 0.21 nM up to 50 nM of α- and P-pheromone (Fig. S5). Concentration determination of α- and P-pheromone in supernatants of *B. subtilis* WD800N P*_liaI_*-*ybxI*-P_H_ for P-pheromone and P*_liaI_*-*yfkD*-α_H_ for α-pheromone, respectively, are shown in Fig. 7. One hour after induction with bacitracin, 43 nM P-pheromone could be determined in supernatants of strain P*_liaI_*-*ybxI*-P_H,_ while strain P*_liaI_*-*yfkD*-α_H_ secreted 8 nM of α-pheromone. Even though secreted pheromone peptide concentration is low compared to industry standards, production was repeatable and robust in *B. subtilis*. This lets us conclude that the developed evaluation vector and the SP toolbox have proven to be useful for heterologous protein secretion in *B. subtilis*.

## 4 Discussion

*B. subtilis* has already been used as a workhorse of White Biotechnology for a long time. Wide application of *B. subtilis* was secured due to its GRAS (generally recognised as safe) status and ability to secrete high amounts of enzymes in relatively cheap culture media.^4^ However, Fu et al., 2018 highlight that a universal SP for protein secretion does not exist, leaving library construction and high-throughput screening approaches the only way to identify, boost expression and render secretion for POI production via SPs.^9^.

In this work, we wanted to develop a faster way to find the optimal SP for protein secretion in *B. subtilis*. Therefore, we established the SP toolbox, including an evaluation vector and 74 SPs. The evaluation vector based on the backbone vector pBS1C^14^ proved to be a feasible and easy-to-use vector in this study. The SP toolbox offers a shotgun approach for high-throughput screening to identify the optimal SP for the POI secretion. Furthermore, the advantage of the developed toolbox here is the design in which SPs were fused with optimised ribosome binding site, spacer and start codon from *B. subtilis* within the storage vector. In future, a toolbox can be easily expanded and more SPs can be added to test their secretion capacities. In this work, eight out of 74 SP are successfully secreting the P-pheromone. The identified SPs responsible for P-pheromone secretion do not have any observable pattern in common. Random correlation between SPs and secreted protein was already observed previously.^9^, ^11^ Unpredictability and unavailability of secretion standardisation (*in silico* prediction tools) are the reasons for the development of the toolbox in this study.

It is worth noticing the two amino acid linkers consisting of threonine and glycine between SPs and the protein of interest, which is a product of restriction cloning with enzymes *Age*I and NgoMIV. Two-amino acid linker was removed in Fu et al., 2018, due to the hypothesis that additional residues influence the cleavage efficiency of SPe for protein secretion.^9^ However, we didn’t test the influence of two amino acid residues on the secretion efficiency because of the wide use of restriction cloning in the development of similar SP libraries.^11^.

By using the newly developed toolbox for secretion of heterologous expressed proteins and peptides, we were able to identify eight SPs for effective P-pheromone secretion, whereas three SPs out of 74 were responsible for α-pheromone secretion. Interestingly, all 11 SPs (see sequence in table S6) vary in their length and share the characteristic α-helix structure of the signal peptide. The structure of the signal peptides can be seen by using the database *Subti*Wiki.^26^ Additionally, after identifying SPs necessary for pheromone secretion, secretion can be maximised through directed evolution and high-throughput screening, as in Shi et al., 2025. Furthermore, we utilised the well-characterised antibiotic-inducible promoter P*_liaI_* to optimise peptide secretion in the *B. subtilis*.^23^, ^24^, ^27^ High inducibility of P*_liaI_* with the antibiotic bacitracin boosts peptide expression within the exponential growth phase and could prevent or reduce the degradation process of secreted peptides by growth-dependent accumulation of extracellular proteases (*e. g*. Bpr, Npr, Epr, or Vpr) of *B. subtilis*. To circumvent the mentioned degradation problem, we also exchanged the production host strain from wild-type to the protease-deficient strain *B. subtilis* WB800N that shows an 8-fold lower protease activity.^19^ As expected, we observed a higher peptide concentration when strain *B. subtilis* WB800N was used for overexpression and secretion of P-pheromone. Since *B. subtilis* WB800N is deficient for only a fractional number of available proteases in *B. subtilis*^8^ we assumed that protease activity is still present and will lower the concentration of expressed and secreted P- and α-pheromones. Here, specialised degradation proteases like Clp, ClpQ or Lon, or protein quality control factors like HtrA and HtrB can contribute to degrade heterologously expressed peptides.^8^, ^28^, ^29^ Improvement in protein expression and preventing degradation of expressed proteins and peptides can be achieved by deleting additional protease-encoding genes from strain *B. subtilis* WB800N. An alternative strategy is to use ‘midi*Bacillus*’ or ‘mini*Bacillus*’ strains.^30^, ^31^ These strains lacking around 36 % of the genome and almost all proteases are characterised by giving the advantage to the production of “difficult-to-produce proteins” but not yet small peptides. However, in case of the expression and secretion of α-pheromone, additional removal of more proteases from the host strain will be most beneficial due to the fact that the 13 amino acid long α-peptide is active as an unfolded protein.^32^ Unfolded secreted proteins are targets for *B. subtilis* extracytoplasmic folding catalysts and quality control proteases, which efficiently remove misfolded or incompletely synthesised proteins.^8^ Unfolded state of α-pheromone and weak secondary structure of P-pheromone is also a reason why we choose Sec secretion system and not TAT-pathway. The TAT pathway is used for the secretion of fully folded proteins with tightly bound cofactors, while proteins secreted by the Sec pathway are unfolded.^33^

In summary, efficient protein expression and secretion in *B. subtilis* is easily possible by using our developed SP-toolbox. Furthermore, the toolbox represents a strategically important application to circumvent a main problem in protein secretion: lack of knowledge regarding the optimal and functional SP essential for effective secretion. In the future, SP toolbox can be improved by expanding it and adding more SPs (up to 173 available in *B. subtilis*, Fu et al., 2018) to the library or developing the protease-free strain of *B. subtilis*.^9^

## CRediT authorship contribution statement

**Tomislav Vološen:** Writing – review & editing, Writing – original draft, Visualization, Validation, Methodology, Investigation, Formal analysis, Data curation. **Henri Max Deda:** Writing – review & editing, Validation, Software, Investigation, Formal analysis, Data curation. **Anica Walther:** Writing – review & editing, Validation, Resources, Methodology, Investigation. **Philipp F. Popp:** Writing – review & editing, Supervision, Project administration, Funding acquisition, Formal analysis. **Thorsten Mascher:** Writing – review & editing, Supervision, Project administration, Conceptualization. **Diana Wolf:** Writing – review & editing, Writing – original draft, Validation, Supervision, Project administration, Funding acquisition, Conceptualization.

## Declaration of competing interest

The authors declare the following financial interests/personal relationships which may be considered as potential competing interests: Diana Wolf reports financial support was provided by European Social Fund. Tomislav Volosen reports financial support was provided by European Social Fund. Henri Max Deda reports financial support was provided by iGEM Foundation. Anica Walther reports administrative support and writing assistance were provided by European Social Fund. Philipp F. Popp reports administrative support and writing assistance were provided by iGEM Foundation. Thorsten Mascher reports administrative support was provided by iGEM Foundation. If there are other authors, they declare that they have no known competing financial interests or personal relationships that could have appeared to influence the work reported in this paper.
