# Supplementary material for: A novel signal peptide toolbox for optimized heterologous expression of yeast pheromones in Bacillus subtilis
Source: J Genet Eng Biotechnol. 2025 Aug 9;23(3):100551. doi: 10.1016/j.jgeb.2025.100551 (PMC12357138; doi:10.1016/j.jgeb.2025.100551)
Supplement: Supplementary Data 1 [file mmc1.docx]

Supplemental Information: Manuscript Heterologous expression of yeast pheromones in *Bacillus* *subtilis* by using a novel signal peptide toolbox

Table S1 Strains used for evaluation vector development

| Plasmid | Description | Source/Reference |
| --- | --- | --- |
| iG17E019 | *E. coli* DH10β pBS1C-[RFP]_wo-*Bsa*I | This study |
| iG17E042 | *E. coli* DH10β pBS1CP*_xylA_*-[RFP]_wo-*Bsa*I | This study |
| iG17E099 | *E. coli* DH10β pSB1C3-[RFPsyn] | This study |
| TME1790 | *E. coli* DH5α pSB1C3-Bba_I732902 (*lacZ*α) | Laboratory stock |
| V174 | *E. coli* DH10β pBS1C, empty plasmid, integ. in *amyE*, amp^r^, cm^r^ | Radeck et al., 2013 |
| V180 | *E. coli* pSB1C3, BBa_K823055iGEM | iGEM, parts registry |
| V246 | *E. coli* DH10β pBS2EP*_xylA_* (9A’s) | Popp et al., 2017 |
| V293 | *E. coli* DH10β pBS1CP*_xylA_*-[P*_lacI_*-RFPsyn2-*lacZ*α], evaluation vector | This study |

Table S2 Oligonucleotides used for the evaluation vector construction

| Primer | Description | Primer sequence |
| --- | --- | --- |
| iG17P051 | PxylA fwd | GATCGAATTCGCGGCCGCTTCTAGAGAAGGCCAAAAAACTGCTGCC |
| iG17P052 | PxylA rev | GATCGCTAGCGAGACCTTCGATAAGCTTGGGATCCC |
| iG17P055 | LacZalpha fwd | GATCACCGGTGCCGGCGCGCAACGCAATTAATGTGAG |
| iG17P056 | LacZalpha rev | GATCCTGCAGCGGCCGCTACTAGTATATAAACGCAGAAAGGCCCAC |
| iG17P110 | RFPsyn_RFC10 rev | GATCTCTGCAGCGGCCGCTACTAGTATTAACCGGTTTAAGCACCTGTACTATGG |
| iG17P159 | RFP-syn-XbaI fwd | TCTAGAGTACTAGAGAAAGAGGAGAAATACTAGATGGCCTCTTCCGAAGATG |
| TM2895 | PliaI fwd EcoRI NotI XbaI | GATCGAATTCGCGGCCGCTTCTAGAGATTGGCCAAGCAGAAAGGTCC |
| TM2896 | PliaI rev SpeI | GATCACTAGTATCGTTTTCCTTGTCTTCATCTTATAC |
| TM3163 | pAH_BsaI_7622_mut_fwd | GGAGCCGGTGAGCGTGGGTCACGCGGTATCATTGCAGC |
| TM3164 | pAH_BsaI_7622_mut_rev | GCTGCAATGATACCGCGTGACCCACGCTCACCGGCTCC |

Table S3 Oligonucleotides used for the signal peptide toolbox development

| Primer | Description | Primer sequence |
| --- | --- | --- |
| TM4487 | BioBrick_prefix_fwd | gatcgaattcgcggccgcttctaga |
| iG17P039 | BioBrick_suffix_rev | gatcctgcagcggccgctactagt |
| iG17P001 | AmyE_SP fwd | gatcgaattcgcggccgcttctagataaggaggtcaaaaatgtttgcaaaacgattcaaaac |
| iG17P002 | AmyE_SP rev | gatctctgcagcggccgctactagtattaaccggtagcactcgcagccgccggtc |
| iG17P003 | AspB_SP fwd | gatcgaattcgcggccgcttctagataaggaggtcaaaaatgaaactggcaaaaagagtatc |
| iG17P004 | AspB_SP rev | gatctctgcagcggccgctactagtattaaccggtcgctttcgctgtgattgccag |
| iG17P005 | bglS_SP fwd | gatcgaattcgcggccgcttctagataaggaggtcaaaaatgccttatctgaaacgagtg |
| iG17P006 | bglS_SP rev | gatctctgcagcggccgctactagtattaaccggtagctgaggcagtagcagtgac |
| iG17P007 | Bpr_SP fwd | gatcgaattcgcggccgcttctagataaggaggtcaaaaatgaggaaaaaaacgaaaaacag |
| iG17P008 | Bpr_SP rev | gatctctgcagcggccgctactagtattaaccggttgccccggctgctcccggaaa |
| iG17P009 | CccA_SP fwd | gatcgaattcgcggccgcttctagataaggaggtcaaaaatgaaatggaacccgcttattc |
| iG17P010 | CccA_SP rev | gatctctgcagcggccgctactagtattaaccggttccttttactgataaaaagaaag |
| iG17P011 | mdh_SP fw | gatcgaattcgcggccgcttctagataaggaggtcaaaaatgggaaatactcgtaaaaaagtttc |
| iG17P012 | mdh_SP rev | gatctctgcagcggccgctactagtattaaccggtaacgtctgccagctctttttg |
| iG17P013 | Csn_SP fwd | gatcgaattcgcggccgcttctagataaggaggtcaaaaatgaaaatcagtatgcaaaaagcag |
| iG17P014 | Csn_SP rev | gatctctgcagcggccgctactagtattaaccggtcgcaaaaaccgtttcgctcatc |
| iG17P015 | DacB_SP fwd | gatcgaattcgcggccgcttctagataaggaggtcaaaaatgcgcattttcaaaaaagc |
| iG17P016 | DacB_SP rev | gatctctgcagcggccgctactagtattaaccggtagcatgtgctgtattcacatt |
| iG17P017 | DacF_SP fwd | gatcgaattcgcggccgcttctagataaggaggtcaaaaatgaaacgtcttttatccactttg |
| iG17P018 | DacF_SP rev | gatctctgcagcggccgctactagtattaaccggttgcaaatgcagacggtgcaa |
| iG17P019 | DltD_SP fwd | gatcgaattcgcggccgcttctagataaggaggtcaaaaatgaaaaagcgttttttcggtc |
| iG17P020 | DltD_SP rev | gatctctgcagcggccgctactagtattaaccggttgcgatggcgcctgcgaatag |
| iG17P071 | Epr_SP fwd | gatcgaattcgcggccgcttctagataaggaggtcaaaaatgaaaaacatgtcttgcaaac |
| iG17P072 | Epr_SP rev | gatctctgcagcggccgctactagtattaaccggtcgcatgagcgagagggcctatg |
| iG17P073 | FliL_SP fwd | gatcgaattcgcggccgcttctagataaggaggtcaaaaatgaagaaaaagttaatgatcatattac |
| iG17P074 | FliL_SP rev | gatctctgcagcggccgctactagtattaaccggtagccgccgccccgagagcac |
| iG17P075 | FliZ_SP fwd | gatcgaattcgcggccgcttctagataaggaggtcaaaaatgaaaaagagtcaatattttattg |
| iG17P076 | FliZ_SP rev | gatctctgcagcggccgctactagtattaaccggttgccgcggcagcagcaatcg |
| iG17P077 | GlpQ_SP fwd | gatcgaattcgcggccgcttctagataaggaggtcaaaaatgagaaaaaatagaatactggc |
| iG17P078 | GlpQ_SP rev | gatctctgcagcggccgctactagtattaaccggttgccgacactggcgttaccataaatg |
| iG17P079 | LipA_SP fwd | gatcgaattcgcggccgcttctagataaggaggtcaaaaatgaaatttgtaaaaagaaggatc |
| iG17P080 | LipA_SP rev | gatctctgcagcggccgctactagtattaaccggtagcggcttttgctgacggctg |
| iG17P081 | LipB_SP fwd | gatcgaattcgcggccgcttctagataaggaggtcaaaaatgaaaaaagtacttatggc |
| iG17P082 | LipB_SP rev | gatctctgcagcggccgctactagtattaaccggtagcttttgcgccagacggcg |
| iG17P083 | LytB_SP fwd | gatcgaattcgcggccgcttctagataaggaggtcaaaaatgaaatcttgcaaacaattg |
| iG17P084 | LytB_SP rev | gatctctgcagcggccgctactagtattaaccggttgcaaaagaaactgatggaatc |
| iG17P085 | LytC_SP fwd | gatcgaattcgcggccgcttctagataaggaggtcaaaaatgcgttcttatataaaagtcc |
| iG17P086 | LytC_SP rev | gatctctgcagcggccgctactagtattaaccggtggccaaagctgttggcacaaaaag |
| iG17P087 | LytD_SP fwd | gatcgaattcgcggccgcttctagataaggaggtcaaaaatgaaaaagagactaatcgc |
| iG17P088 | LytD_SP rev | gatctctgcagcggccgctactagtattaaccggttgcctgggcagaaccagacatg |
| iG17P089 | LytF_SP fwd | gatcgaattcgcggccgcttctagataaggaggtcaaaaatgaaaaatgaaaaagaaattagcagcag |
| iG17P090 | LytF_SP rev | gatctctgcagcggccgctactagtattaaccggttgcttcagctggtgtcactac |
| iG17P091 | LytR_sp fwd | gatcgaattcgcggccgcttctagataaggaggtcaaaaatgagaaacgaacgcagaaaaaag |
| iG17P092 | LytR_sp rev | gatctctgcagcggccgctactagtattaaccggtagctgctttatgccataagtag |
| iG17P093 | Mdr_SP fwd | gatcgaattcgcggccgcttctagataaggaggtcaaaaatggacacaacaacagcaaaac |
| iG17P094 | Mdr_SP rev | gatctctgcagcggccgctactagtattaaccggtcgcggtggcaacaatcgtattg |
| iG17P095 | MotB_SP fwd | gatcgaattcgcggccgcttctagataaggaggtcaaaaatggcgagaaaaaagaagaag |
| iG17P096 | MotB_SP rev | gatctctgcagcggccgctactagtattaaccggtgctgctcgcgtacagcacaat |
| iG17P097 | Mpr_SP fwd | gatcgaattcgcggccgcttctagataaggaggtcaaaaatgaaattagttccaagattcag |
| iG17P098 | Mpr_SP rev | gatctctgcagcggccgctactagtattaaccggtcgcttttgccggtacgccaaaag |
| iG17P099 | MreC_SP fwd | gatcgaattcgcggccgcttctagataaggaggtcaaaaatgccgaataagcggttaatg |
| iG17P100 | MreC_SP rev | gatctctgcagcggccgctactagtattaaccggtgaaaatccaatcatagccacc |
| iG17P101 | NprE_Sp fwd | gatcgaattcgcggccgcttctagataaggaggtcaaaaatgggtttaggtaagaaattgtc |
| iG17P102 | NprE_Sp rev | gatctctgcagcggccgctactagtattaaccggtagcctgaacacctggcagg |
| iG17P103 | pbpB_SP fwd | gatcgaattcgcggccgcttctagataaggaggtcaaaaatgattcaaatgccaaaaaag |
| iG17P104 | pbpB_SP rev | gatctctgcagcggccgctactagtattaaccggttgccattctccccaggatg |
| iG17P105 | PbpD_SP fwd | gatcgaattcgcggccgcttctagataaggaggtcaaaaatgaccatgttacgaaaaataatc |
| iG17P106 | PbpD_SP rev | gatctctgcagcggccgctactagtattaaccggtagcgataactgtaaatgcaaac |
| iG17P107 | PdpX_SP fwd | gatcgaattcgcggccgcttctagataaggaggtcaaaaatgacaagcccaacccgcag |
| iG17P108 | PdpX_SP rev | gatctctgcagcggccgctactagtattaaccggtagcattccaaatcgtaatgcaaacc |
| iG17P113 | RpmG_SP fwd | gatcgaattcgcggccgcttctagataaggaggtcaaaaatgagaaaaaagattacgttagcat |
| iG17P114 | RpmG_SP rev | gatctctgcagcggccgctactagtattaaccggtcgctgatgcagagctctt |
| iG17P115 | SacB_SP fwd | gatcgaattcgcggccgcttctagataaggaggtcaaaaatgaacatcaaaaagtttgcaaaac |
| iG17P116 | SacB_SP rev | gatctctgcagcggccgctactagtattaaccggtcgcaaacgcttgagttgc |
| iG17P117 | SacC_SP fwd | gatcgaattcgcggccgcttctagataaggaggtcaaaaatgaaaaagagactgattcaagtca |
| iG17P118 | SacC_SP rev | gatctctgcagcggccgctactagtattaaccggttgcatctgccgaaaatgc |
| iG17P119 | SleB_SP fwd | gatcgaattcgcggccgcttctagataaggaggtcaaaaatgaagtccaaaggatcgattatgg |
| iG17P120 | SleB_SP rev | gatctctgcagcggccgctactagtattaaccggtcgaaaaggcagagatcgtttc |
| iG17P121 | SpoIID_SP fwd | gatcgaattcgcggccgcttctagataaggaggtcaaaaatgaaacaattcgcaatcacactat |
| iG17P122 | SpoIID_SP rev | gatctctgcagcggccgctactagtattaaccggtggcccccgcttccttatt |
| iG17P123 | SpoIIP_SP fwd | gatcgaattcgcggccgcttctagataaggaggtcaaaaatgagaaataaacgcagaaacagac |
| iG17P124 | SpoIIP_SP rev | gatctctgcagcggccgctactagtattaaccggtcacaccggatagaacaaaaatca |
| iG17P125 | SpoIIQ_SP fwd | gatcgaattcgcggccgcttctagataaggaggtcaaaaatgagagaggaagaaaagaaaactt |
| iG17P126 | SpoIIQ_SP rev | gatctctgcagcggccgctactagtattaaccggtaaggacagctgttaaaatgacg |
| iG17P127 | SpoIIR_SP fwd | gatcgaattcgcggccgcttctagataaggaggtcaaaaatgaaaaaaacagtaatcatttgta |
| iG17P128 | SpoIIR_SP rev | gatctctgcagcggccgctactagtattaaccggttacgagcgctccggataa |
| iG17P129 | Vpr_SP fwd | gatcgaattcgcggccgcttctagataaggaggtcaaaaatgaaaaaggggatcattcgctttct |
| iG17P130 | Vpr_SP rev | gatctctgcagcggccgctactagtattaaccggtagccggagctgcctgaac |
| iG17P131 | TyrA_SP fwd | gatcgaattcgcggccgcttctagataaggaggtcaaaaatgaatcaaatgaaagatacaatat |
| iG17P132 | TyrA_SP rev | gatctctgcagcggccgctactagtattaaccggtggctagggcaatcgaacc |
| iG17P133 | Pel_SP fwd | gatcgaattcgcggccgcttctagataaggaggtcaaaaatgaaaaaagtgatgttagctac |
| iG17P134 | Pel_SP rev | gatctctgcagcggccgctactagtattaaccggttgcgttcgcgccagctggag |
| iG17P135 | PelB_SP fwd | gatcgaattcgcggccgcttctagataaggaggtcaaaaatgaaacgactttgtttatgg |
| iG17P136 | PelB_SP rev | gatctctgcagcggccgctactagtattaaccggtacctaatgcttttccaggcaag |
| iG17P137 | PenP_SP fwd | gatcgaattcgcggccgcttctagataaggaggtcaaaaatgaagttgaaaactaaagcg |
| iG17P138 | PenP_SP rev | gatctctgcagcggccgctactagtattaaccggttgcttcggcatgtgttgag |
| iG17P139 | PhoA_SP fwd | gatcgaattcgcggccgcttctagataaggaggtcaaaaatgaaaaaaatgagtttgtttc |
| iG17P140 | PhoA_SP rev | gatctctgcagcggccgctactagtattaaccggtagctccggcaaagattccag |
| iG17P141 | PhoB_SP fwd | gatcgaattcgcggccgcttctagataaggaggtcaaaaatgaaaaaattcccgaagaaa |
| iG17P142 | PhoB_SP rev | gatctctgcagcggccgctactagtattaaccggtggcgctggcttcaggcacac |
| iG17P143 | PhrA_SP fwd | gatcgaattcgcggccgcttctagataaggaggtcaaaaatgaaatctaaatggatgtcag |
| iG17P144 | PhrA_SP rev | gatctctgcagcggccgctactagtattaaccggttgcatgaaccatcacctgag |
| iG17P145 | PhrC_SP fwd | gatcgaattcgcggccgcttctagataaggaggtcaaaaatgaaattgaaatctaagttgtt |
| iG17P146 | PhrC_SP rev | gatctctgcagcggccgctactagtattaaccggtcgcattagcagaaacgccag |
| iG17P147 | PhrF_SP fwd | gatcgaattcgcggccgcttctagataaggaggtcaaaaatgaaattgaagtctaaactattac |
| iG17P148 | PhrF_SP rev | gatctctgcagcggccgctactagtattaaccggttgcaatagttgttgccacgaac |
| iG17P149 | PhrG_SP fwd | gatcgaattcgcggccgcttctagataaggaggtcaaaaatgaaaagatttctgattggc |
| iG17P150 | PhrG_SP rev | gatctctgcagcggccgctactagtattaaccggtcgcaataaaccaacctgata |
| iG17P151 | PhrK_SP fwd | gatcgaattcgcggccgcttctagataaggaggtcaaaaatgaaaaaacttgtgctttg |
| iG17P152 | PhrK_SP rev | gatctctgcagcggccgctactagtattaaccggtagctactccacttaaaatcac |
| iG17P160 | WapA_SP fwd | gatcgaattcgcggccgcttctagataaggaggtcaaaaatgaaaaaaagaaagaggcgaaact |
| iG17P161 | WapA_SP rev | gatctctgcagcggccgctactagtattaaccggttgctagtacatcggctgg |
| iG17P162 | YbbC_SP fwd | gatcgaattcgcggccgcttctagataaggaggtcaaaaatgagaaaaacaatattcgcttttc |
| iG17P163 | YbbC_SP rev | gatctctgcagcggccgctactagtattaaccggtagcagaggcagcggtta |
| iG17P164 | YbbE_SP fwd | gatcgaattcgcggccgcttctagataaggaggtcaaaaatgaaaacaaagacactgttcatatt |
| iG17P165 | YbbE_SP rev | gatctctgcagcggccgctactagtattaaccggttgcgaaggtttcatttggc |
| iG17P166 | YbbR_SP fwd | gatcgaattcgcggccgcttctagataaggaggtcaaaaatggataaattcttaaacaaccgct |
| iG17P167 | YbbR_SP rev | gatctctgcagcggccgctactagtattaaccggtgctgttaaccgccacataaagc |
| iG17P168 | YbdG_SP fwd | gatcgaattcgcggccgcttctagataaggaggtcaaaaatgaaaacattatggaaagtcctca |
| iG17P169 | YbdG_SP rev | gatctctgcagcggccgctactagtattaaccggtcgagacggatacaagcaaaaccaaa |
| iG17P170 | YbdN_SP fwd | gatcgaattcgcggccgcttctagataaggaggtcaaaaatggtgaaaaaatggcttattcaat |
| iG17P171 | YbdN_SP rev | gatctctgcagcggccgctactagtattaaccggtagctgatgccgaatacgtaaag |
| iG17P172 | YbfO_SP fwd | gatcgaattcgcggccgcttctagataaggaggtcaaaaatgaaacgaatgatagtgagaatgac |
| iG17P173 | YbfO_SP rev | gatctctgcagcggccgctactagtattaaccggtagcacgggctgaggctgaaaaagaa |
| iG17P174 | YbxI_SP fwd | gatcgaattcgcggccgcttctagataaggaggtcaaaaatgaaaaaatggatatatgttgtgc |
| iG17P175 | YbxI_SP rev | gatctctgcagcggccgctactagtattaaccggttgcgtggacggagaagc |
| iG17P176 | YdbK_SP fwd | gatcgaattcgcggccgcttctagataaggaggtcaaaaatgaaactttttaatcggaaggtca |
| iG17P177 | YdbK_SP rev | gatctctgcagcggccgctactagtattaaccggtactgatgacaatccgtttaatgatc |
| iG17P178 | YdhT_SP fwd | gatcgaattcgcggccgcttctagataaggaggtcaaaaatgtttaagaaacatacgatctcttt |
| iG17P179 | YdhT_SP rev | gatctctgcagcggccgctactagtattaaccggttgctaaaacagcagacgc |
| iG17P180 | YdjM_SP fwd | gatcgaattcgcggccgcttctagataaggaggtcaaaaatgttgaagaaagtcattttagc |
| iG17P181 | YdjM_SP rev | gatctctgcagcggccgctactagtattaaccggtcgcactggcatctgatgaaaaac |
| iG17P182 | YdjN_SP fwd | gatcgaattcgcggccgcttctagataaggaggtcaaaaatgaaaaaaagaatcatattattattagc |
| iG17P183 | YdjN_SP rev | gatctctgcagcggccgctactagtattaaccggtcgcaacccccgcggcagctgc |
| iG17P184 | YfhK_SP fwd | gatcgaattcgcggccgcttctagataaggaggtcaaaaatgaaaaagaaacaagtaatgc |
| iG17P185 | YfhK_SP rev | gatctctgcagcggccgctactagtattaaccggtagcttttgctgcgggagcgg |
|  |  |  |
| iG17P186 | YfjS/(pdaA)_SP fwd | gatcgaattcgcggccgcttctagataaggaggtcaaaaatgaagtggatgtgttcaatatg |
| iG17P187 | YfjS/(pdaA)_SP rev | gatctctgcagcggccgctactagtattaaccggtcgcctgtgctgcacctccggc |
| iG17P188 | YfkD_SP fwd | gatcgaattcgcggccgcttctagataaggaggtcaaaaatgatgaaaaagctatttcattcc |
| iG17P189 | YfkD_SP rev | gatctctgcagcggccgctactagtattaaccggtcgcgtggatgggctgaacgccg |
| iG17P190 | YfkN_SP fwd | gatcgaattcgcggccgcttctagataaggaggtcaaaagtgagaatacagaaaagacgaac |
| iG17P191 | YfkN_SP rev | gatctctgcagcggccgctactagtattaaccggttgcatgaatgggtggtgttggga |
| iG17P192 | YhcR_SP fwd | gatcgaattcgcggccgcttctagataaggaggtcaaaaatgctgtctgtcgaaatgata |
| iG17P193 | YhcR_SP rev | gatctctgcagcggccgctactagtattaaccggtagcttcgaacgtgtacattac |
| iG17P194 | YhdC_SP fwd | gatcgaattcgcggccgcttctagataaggaggtcaaaaatgaaatccctgccgtatac |
| iG17P195 | YhdC_SP rev | gatctctgcagcggccgctactagtattaaccggtagccattgacacgataatcaa |
| iG17P196 | YhfM_SP fwd | gatcgaattcgcggccgcttctagataaggaggtcaaaaatgaaaaaaatagtggcagcc |
| iG17P197 | YhfM_SP rev | gatctctgcagcggccgctactagtattaaccggtcgcgtctaccgattgatacac |
| iG17P198 | YhjA_SP fwd | gatcgaattcgcggccgcttctagataaggaggtcaaaaatgaaaaaagcggcggcg |
| iG17P199 | YhjA_SP rev | gatctctgcagcggccgctactagtattaaccggtggcttccgccacatggccag |
| iG17P201 | YjcN_SP_fwd | gatcgaattcgcggccgcttctagataaggaggtcaaaaatgaaaaagaaaactaaaattatactttctctc |
| iG17P202 | YjcN_SP_rev | gatctctgcagcggccgctactagtattaaccggtcgaagaagctgtaaagacaac |
| iG17P203 | YjdB_SP_fwd | gatcgaattcgcggccgcttctagataaggaggtcaaaaatgaatttcaaaaaaacggttgtttctgc |
| iG17P204 | YjdB_SP_rev | gatctctgcagcggccgctactagtattaaccggtagctgaagctaccccgct |
| iG17P205 | YjfA_SP_fwd | gatcgaattcgcggccgcttctagataaggaggtcaaaattgaaaagactgtttatgaaggcttcatt |
| iG17P206 | YjfA_SP_rev | gatctctgcagcggccgctactagtattaaccggtcgccttggcgggtg |
| iG17P207 | YjiA_SP_fwd | gatcgaattcgcggccgcttctagataaggaggtcaaaagtggcagcacagactgattacaa |
| iG17P208 | YjiA_SP_rev | gatctctgcagcggccgctactagtattaaccggtagagaacacaaagagtacgaatgc |
| iG17P209 | YkoJ_SP_fwd | gatcgaattcgcggccgcttctagataaggaggtcaaaaatgctcaagaaaaaatggatggtcg |
| iG17P210 | YkoJ_SP_rev | gatctctgcagcggccgctactagtattaaccggtggcaaacgcgttgtagctg |
| iG17P211 | YkvV_SP_fwd | gatcgaattcgcggccgcttctagataaggaggtcaaaaatgttgacgaagcgcttgc |
| iG17P212 | YkvV_SP_rev | gatctctgcagcggccgctactagtattaaccggtagcttgtgccgcacctg |
| iG17P213 | YkwD_SP_fwd | gatcgaattcgcggccgcttctagataaggaggtcaaaaatgaagaaagcatttattttatctgct |
| iG17P214 | YkwD_SP_rev | gatctctgcagcggccgctactagtattaaccggtcgctgatgcttgctg |
| iG17P215 | YlaE_SP_fwd | gatcgaattcgcggccgcttctagataaggaggtcaaaaatgaagaaaacatttgtaaaaaaagctatgt |
| iG17P216 | YlaE_SP_rev | gatctctgcagcggccgctactagtattaaccggttgcgctggctgc |
| iG17P217 | YlbL_SP_fwd | gatcgaattcgcggccgcttctagataaggaggtcaaaattgctacgtaaaaaacattttagctggatgc |
| iG17P218 | YlbL_SP_rev | gatctctgcagcggccgctactagtattaaccggtcgcttctcccggttttgtaatatagtat |
| iG17P219 | YlqB_SP_fwd | gatcgaattcgcggccgcttctagataaggaggtcaaaaatgaaaaaaatcggtttattgtttatgtta |
| iG17P220 | YlqB_SP_rev | gatctctgcagcggccgctactagtattaaccggtagcgtctgcttgct |
| iG17P221 | YlxF_SP_fwd | gatcgaattcgcggccgcttctagataaggaggtcaaaaatgtccggcaaaaagaaagaatcag |
| iG17P222 | YlxF_SP_rev | gatctctgcagcggccgctactagtattaaccggtaccagcagcccaaagaacaa |

Table S4 List of plasmids developed for signal peptide toolbox stored in *E. coli* DH10β

| Strain | Description | Source/Reference |
| --- | --- | --- |
| iG17E005 | pSB1C3 - AmyE_SP | This study |
| iG17E006 | pSB1C3-AspB_SP | This study |
| iG17E007 | pSB1C3-BglS_SP | This study |
| iG17E008 | pSB1C3-CccA_SP | This study |
| iG17E009 | pSB1C3-Bpr_SP | This study |
| iG17E010 | pSB1C3-Csn_SP | This study |
| iG17E011 | pSB1C3-DacB_SP | This study |
| iG17E012 | pSB1C3-DacF_SP | This study |
| iG17E013 | pSB1C3-DltD_SP | This study |
| iG17E014 | pSB1C3-CitH_SP | This study |
| iG17E046 | pSB1C3-Epr_SP | This study |
| iG17E047 | pSB1C3-FliL_SP | This study |
| iG17E048 | pSB1C3-FliZ_SP | This study |
| iG17E049 | pSB1C3-GlpQ_SP | This study |
| iG17E050 | pSB1C3-LipA_SP | This study |
| iG17E051 | pSB1C3-LytB_SP | This study |
| iG17E052 | pSB1C3-LytC_SP | This study |
| iG17E053 | pSB1C3-LytD_SP | This study |
| iG17E100 | pSB1C3-YjcN_SP | This study |
| iG17E101 | pSB1C3-YjdB_SP | This study |
| iG17E102 | pSB1C3-YjfA_SP | This study |
| iG17E103 | pSB1C3-YjiA_SP | This study |
| iG17E104 | pSB1C3-YkoJ_SP | This study |
| iG17E105 | pSB1C3-YkvV_SP | This study |
| iG17E106 | pSB1C3-YkwD_SP | This study |
| iG17E107 | pSB1C3-YlaE_SP | This study |
| iG17E108 | pSB1C3-YlbL_SP | This study |
| iG17E109 | pSB1C3-YlqB_SP | This study |
| iG17E110 | pSB1C3-YlxF_SP | This study |
| iG17E122 | pSB1C3 - WapA_SP | This study |
| iG17E123 | pSB1C3 -YbbC_SP | This study |
| iG17E124 | pSB1C3 - YbbE_SP | This study |
| iG17E125 | pSB1C3 - YbbR_SP | This study |
| iG17E126 | pSB1C3 -YbdG_SP | This study |
| iG17E127 | pSB1C3 - YbdN_SP | This study |
| iG17E128 | pSB1C3 -YbfO_SP | This study |
| iG17E129 | pSB1C3 - YbxI_SP | This study |
| iG17E130 | pSB1C3 - YdbK_SP | This study |
| iG17E131 | pSB1C3 -YdhT_SP | This study |
| iG17E132 | pSB1C3 - SpoIIP_SP | This study |
| iG17E133 | pSB1C3 -SpoIID_SP | This study |
| iG17E134 | pSB1C3 -Vpr_SP | This study |
| iG17E135 | pSB1C3 - TyrA_SP | This study |
| iG17E136 | pSB1C3 - SpoIIR_SP | This study |
| iG17E137 | pSB1C3 -SpoIIQ_SP | This study |
| iG17E138 | pSB1C3 - SleB_SP | This study |
| iG17E139 | pSB1C3 - SacC_SP | This study |
| iG17E140 | pSB1C3 - RpmG_SP | This study |
| iG17E141 | pSB1C3 - SacB_SP | This study |
| iG17E142 | pSB1C3-LytR_SP | This study |
| iG17E143 | pSB1C3-Mdr_SP | This study |
| iG17E144 | pSB1C3-Mpr_SP | This study |
| iG17E145 | pSB1C3-NprE_SP | This study |
| iG17E146 | pSB1C3-PbpX_SP | This study |
| iG17E148 | pSB1C3 - YdjM_SP | This study |
| iG17E149 | pSB1C3 - YdjN_SP | This study |
| iG17E150 | pSB1C3 - YfhK_SP | This study |
| iG17E151 | pSB1C3 - YfjS_SP | This study |
| iG17E152 | pSB1C3 - YfkD_SP | This study |
| iG17E153 | pSB1C3 - YfkN_SP | This study |
| iG17E154 | pSB1C3 - YhcR_SP | This study |
| iG17E155 | pSB1C3 - YhdC_SP | This study |
| iG17E156 | pSB1C3 - YhfM_SP | This study |
| iG17E157 | pSB1C3 - YhjA_SP | This study |
| iG17E159 | pSB1C3 - Pel_SP | This study |
| iG17E160 | pSB1C3 - PelB_SP | This study |
| iG17E161 | pSB1C3 - PenP_SP | This study |
| iG17E162 | pSB1C3 - PhoA_SP | This study |
| iG17E163 | pSB1C3 - PhoB_SP | This study |
| iG17E164 | pSB1C3 - PhrA_SP | This study |
| iG17E165 | pSB1C3 - PhrC_SP | This study |
| iG17E166 | pSB1C3 - PhrF_SP | This study |
| iG17E167 | pSB1C3 - PhrG_SP | This study |
| iG17E168 | pSB1C3 - PhrK_SP | This study |

Table S5 Adjusted multi-template PCR reaction

| Reaction setup | | Thermocycling Conditions | | |
| --- | --- | --- | --- | --- |
| Component | Volume (µL) | PCR Step | Temperature (°C) | Time (sec) |
| Milli-Q Water | 44 | Denaturation | 98 | 30 |
| 5X Q5 Reaction Buffer | 20 | Denaturation | 98 | 10 |
| Oligo TM4487 | 15 | Annealing | 63 | 40 |
| Oligo iG178P039 | 15 | Elongation | 72 | 20 |
| Signal Peptide Mix | 3 | Final Extension | 72 | 120 |
| dNTPs | 2 | Hold | 15 | pause |
| Q5 Polymerase | 1 |  |  |  |

Q5 Polymerase and dNTPs were purchased from NEB (New England Biolabs (Ipswich, Massachusetts, US)). Recommended oligonucleotide end concentration is determined as 1.5 µM. 1.5 ng of signal peptide mix per 100 µL reaction volume should not be exceeded. Number of amplifications cycled in thermocycler should not exceed 30.

Table S6 Signal peptide sequence of all 74 proteins.

| Name | Signal peptide sequence |
| --- | --- |
| AmyE | MFAKRFKTSLLPLFAGFLLLFHLVLAGPAAASA |
| AspB | MKLAKRVSALTPSTTLAITAKA |
| BglS | MPYLKRVLLLLVTGLFMSLFAVTATASA |
| Bpr | MRKKTKNRLISSVLSTVVISSLLFPGAAGA |
| CccA | MKWNPLIPFLLIAVLGIGLTFFLSVKG |
| CitH | MGNTRKKVSVIGAGFTGATTAFLIAQKELADV |
| Csn | MKISMQKADFWKKAAISLLVFTMFFTLMMSETVFA |
| DacB | MRIFKKAVFVIMISFLIATVNVNTAHA |
| DacF | MKRLLSTLLIGIMLLTFAPSAFA |
| DltD | MKKRFFGPIILAFILFAGAIA |
| Epr | MKNMSCKLVVSVTLFFSFLTIGPLAHA |
| FliL | MKKKLMIILLIILIVIGALGAAA |
| FliZ | MKKSQYFIVFICFFVLFSVHPIAAAAA |
| GlpQ | MRKNRILALFVLSLGLLSFMVTPVSA |
| LipA | MKFVKRRIIALVTILMLSVTSLFALQPSAKAA |
| LytB | MKSCKQLIVCSLAAILLLIPSVSFA |
| LytC | MRSYIKVLTMCFLGLILFVPTALA |
| LytD | MKKRLIAPMLLSAASLAFFAMSGSAQA |
| LytR | MRNERRKKKKTLLLTILTIIGLLVLGTGGYAYYLWHKAA |
| Mdr | MDTTTAKQASTKFVVLGLLLGILMSAMDNTIVATA |
| Mpr | MKLVPRFRKQWFAYLTVLCLALAAAVSFGVPAKA |
| NprE | MGLGKKLSVAVAASFMSLSISLPGVQA |
| PbpX | MTSPTRRRTAKRRRRKLNKRGKLLFGLLAVMVCITIWNA |
| Pel | MKKVMLATALFLGLTPAGANA |
| PelB | MKRLCLWFTVFSLFLVLLPGKALG |
| PenP | MKLKTKASIKFGICVGLLCLSITGFTPFFNSTHAEA |
| PhoA | MKKMSLFQNMKSKLLPIAAVSVLTAGIFAGA |
| PhoB | MKKFPKKLLPIAVLSSIAFSSLASGSVPEASA |
| PhrA | MKSKWMSGLLLVAVGFSFTQVMVHA |
| PhrC | MKLKSKLFVICLAAAAIFTAAGVSANA |
| PhrF | MKLKSKLLLSCLALSTVFVATTIA |
| PhrG | MKRFLIGAGVAAVILSGWFIA |
| PhrK | MKKLVLCVSILAVILSGVA |
| RpmGB | MRKKITLACKTCGNRNYTTMKSSASA |
| SacB | MNIKKFAKQATVLTFTTALLAGGATQAFA |
| SacC | MKKRLIQVMIMFTLLLTMAFSADA |
| SleB | MKSKGSIMACLILFSFTITTFINTETISAFS |
| SpoIID | MKQFAITLSVLCALILLVPTLLVIPFQHNKEAGA |
| SpoIIP | MRNKRRNRQIVVAVNGGKAVKAIFLFIVSLIVIFVLSGV |
| SpoIIQ | MREEEKKTSQVKKLQQFFRKRWVFPAIYLVSAAVILTAVL |
| SpoIIR | MKKTVIICIYIFLLLSGALV |
| TyrA | MNQMKDTILLAGLGLIGGSIALA |
| Vpr | MKKGIIRFLLVSFVLFFALSTGITGVQAAPA |
| WapA | MKKRKRRNFKRFIAAFLVLALMISLVPADVLA |
| YbbC | MRKTIFAFLTGLMMFGTITAASA |
| YbbE | MKTKTLFIFSAILTLSIFAPNETFA |
| YbbR | MDKFLNNRWAVKIIALLFALLLYVAVNS |
| YbdG | MKTLWKVLKIVFVSLAALVLLVSVS |
| YbdN | MVKKWLIQFAVMLSVLSTFTYSASA |
| YbfO | MKRMIVRMTLPLLIVCLAFSSFSASARA |
| YbxI | MKKWIYVVLVLSIAGIGGFSVHA |
| YdbK | MKLFNRKVTLVSLILMAVFQFFMALIIKRIVIS |
| YdhT | MFKKHTISLLIIFLLASAVLA |
| YdjM | MLKKVILAAFILVGSTLGAFSFSSDASA |
| YdjN | MKKRIILLLAVIIAAAAAGVA |
| YfhK | MKKKQVMLALTAAAGLGLTALHSAPAAKA |
| YfjS | MKWMCSICCAAVLLAGGAAQA |
| YfkD | MMKKLFHSTLIVLLFFSFFGVQPIHA |
| YfkN | MRIQKRRTHVENILRILLPPIMILSLILPTPPIHA |
| YhcR | MLSVEMISRQNRCHYVYKGGNMMRRILHIVLITALMFLNVMYTFEA |
| YhdC | MKSLPYTIALLFCGLIIVSMA |
| YhfM | MKKIVAAIVVIGLVFIAFFYLYSRSGDVYQSVDA |
| YhjA | MKKAAAVLLSLGLVFGFSYGAGHVAEA |
| YjcN | MKKKTKIILSLLAALIVILIVLPVLSPVVFTASS |
| YjdB | MNFKKTVVSALSISALALSVSGVASA |
| YjfA | MKRLFMKASLVLFAVVFVFAVKGAPAKA |
| YjiA | MAAQTDYKKQVVGILLSLAFVLFVFS |
| YkoJ | MLKKKWMVGLLAGCLAAGGFSYNAFA |
| YkvV | MLTKRLLTIYIMLLGLIAWFPGAAQA |
| YkwD | MKKAFILSAAAAVGLFTFGGVQQASA |
| YlaE | MKKTFVKKAMLTTAAMTSAALLTFGPDAASA |
| YlbL | MLRKKHFSWMLVILILIAVLSFIKLPYYITKPGEA |
| YlqB | MKKIGLLFMLCLAALFTIGFPAQQADA |
| YlxF | MSGKKKESGKFRSVLLIIILPLMFLLIAGGIVLWAAG |

Purple mark the signal peptide responsible for P-pheromone secretion; Green mark the signal peptide responsible for α-pheromone secretion.


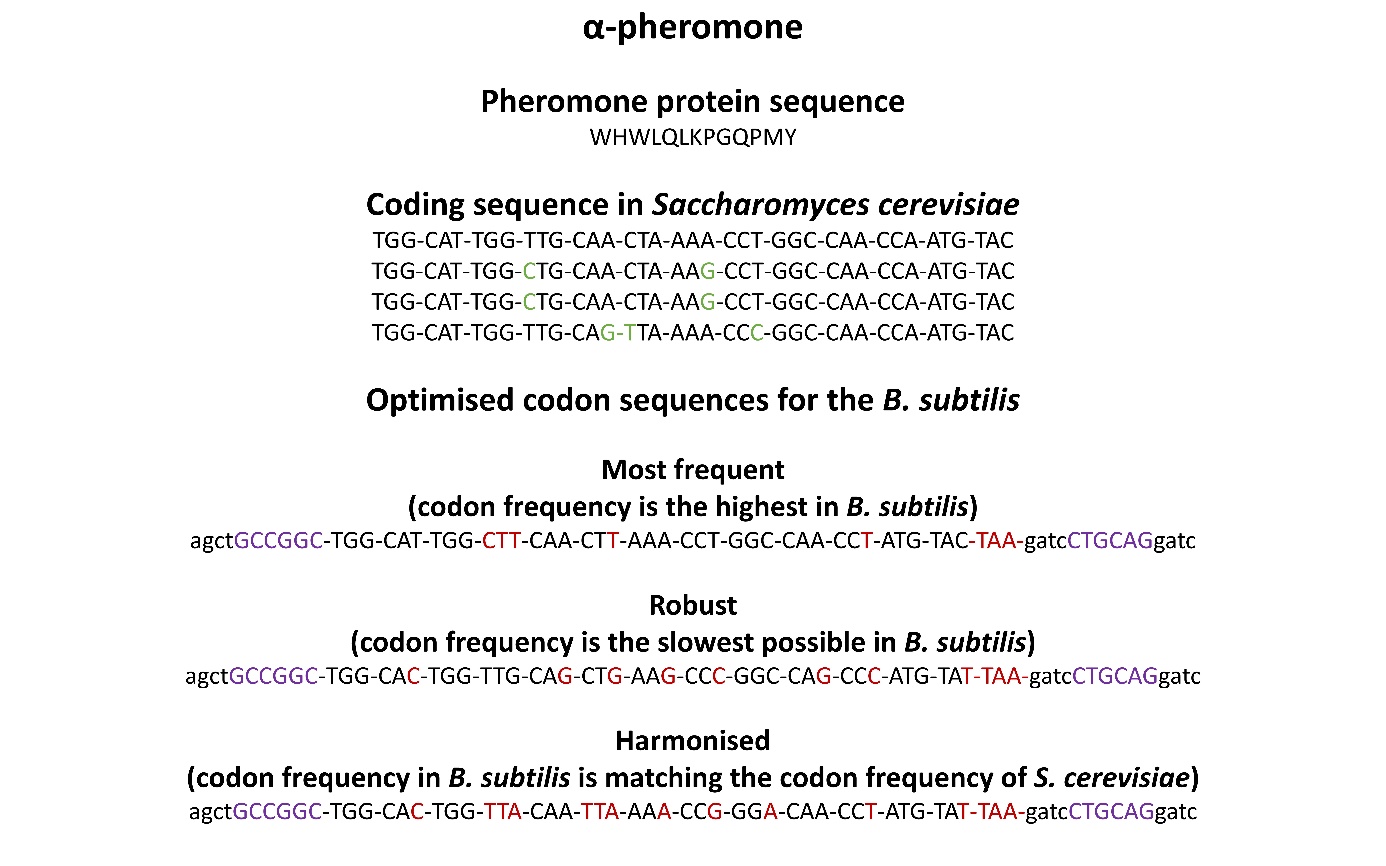


**Figure S1. Sequence of α-pheromone in *S. cerevisae* and codon optimised sequences in *B. subtilis*****.** Purple coloured letters = restriction sites needed for cloning. Red coloured letters = modified codon nucleotides. Green coloured letters = difference in native coding sequence


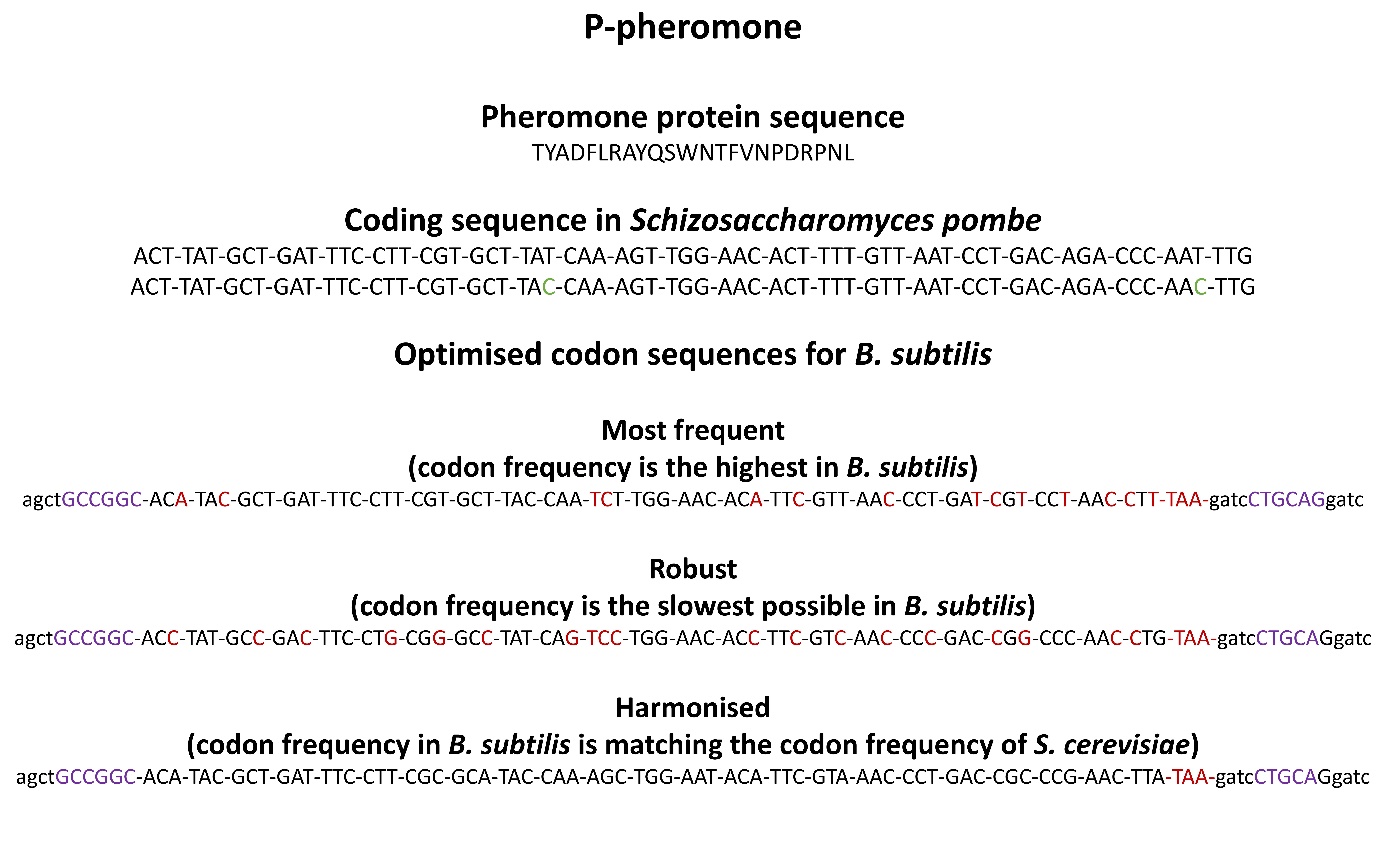


**Figure S2. Sequence of P-pheromones in *S. cerevisiae* and codon optimised sequence in *B. subtilis***. Purple coloured letters = restriction sites needed for cloning. Red coloured letters = modified codon nucleotides. Green coloured letters = difference in native coding sequence.


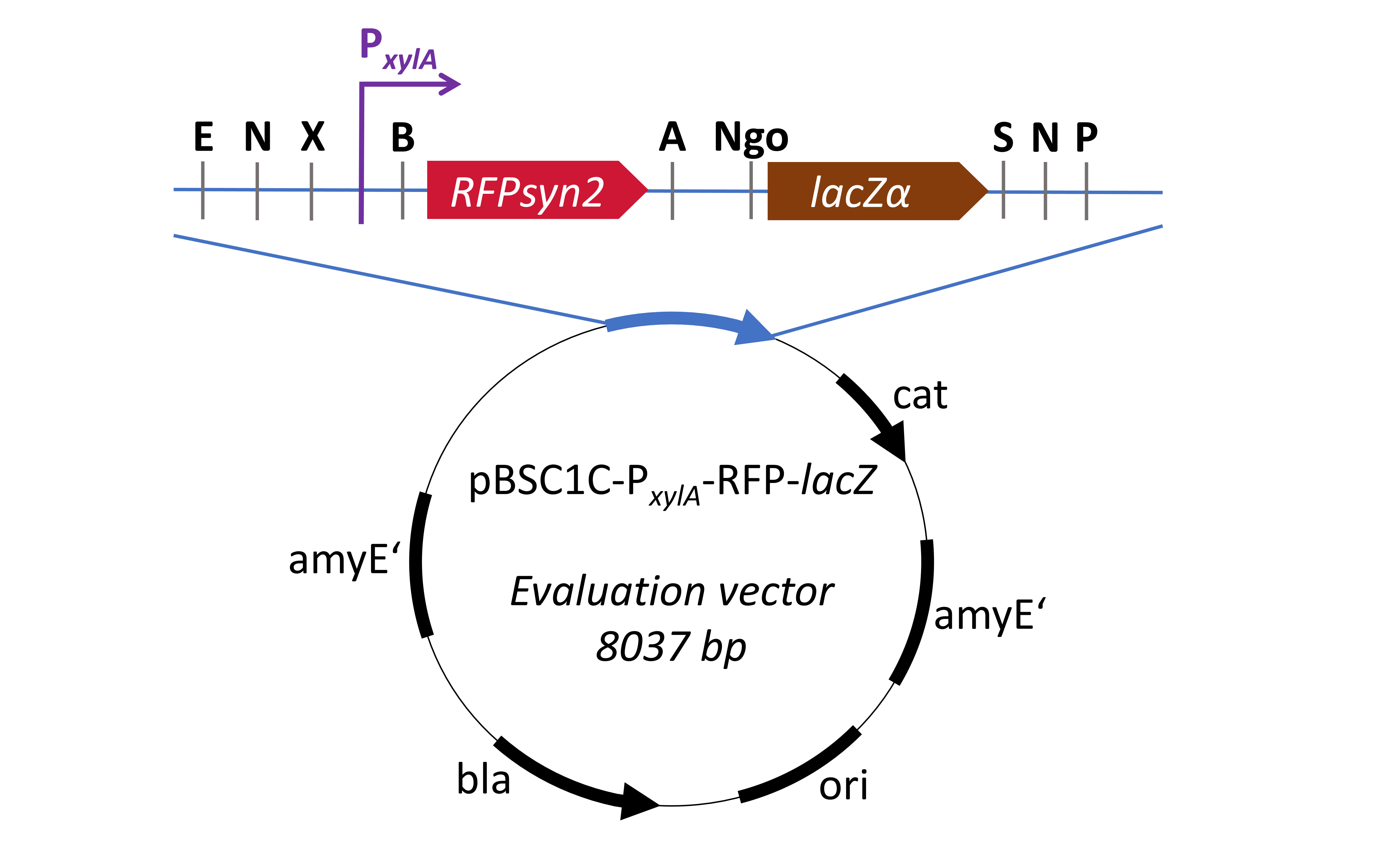


**Figure S3. Evaluation vector map**. Map of the evaluation vector consists of plasmid necessary elements and zoomed representation of multiple cloning site (MCS); cat-chloramphenicol resistance cassette; *amyE*-α-amylase gene locus of *B. subtilis*, ori-origin of replication in *E. coli*, bla-β-lactamase (ampicillin resistance). The multiple cloning site (MCS) fulfil the BioBrick^TM^ standard: E-*Eco*RI, N-*Not*I, X-*Xba*I, B-*Bsa*I (*XbaI* overhang), A-*Age*I, Ngo-*Ngo*MIV, S-*Spe*I, and P-*Pst*I.


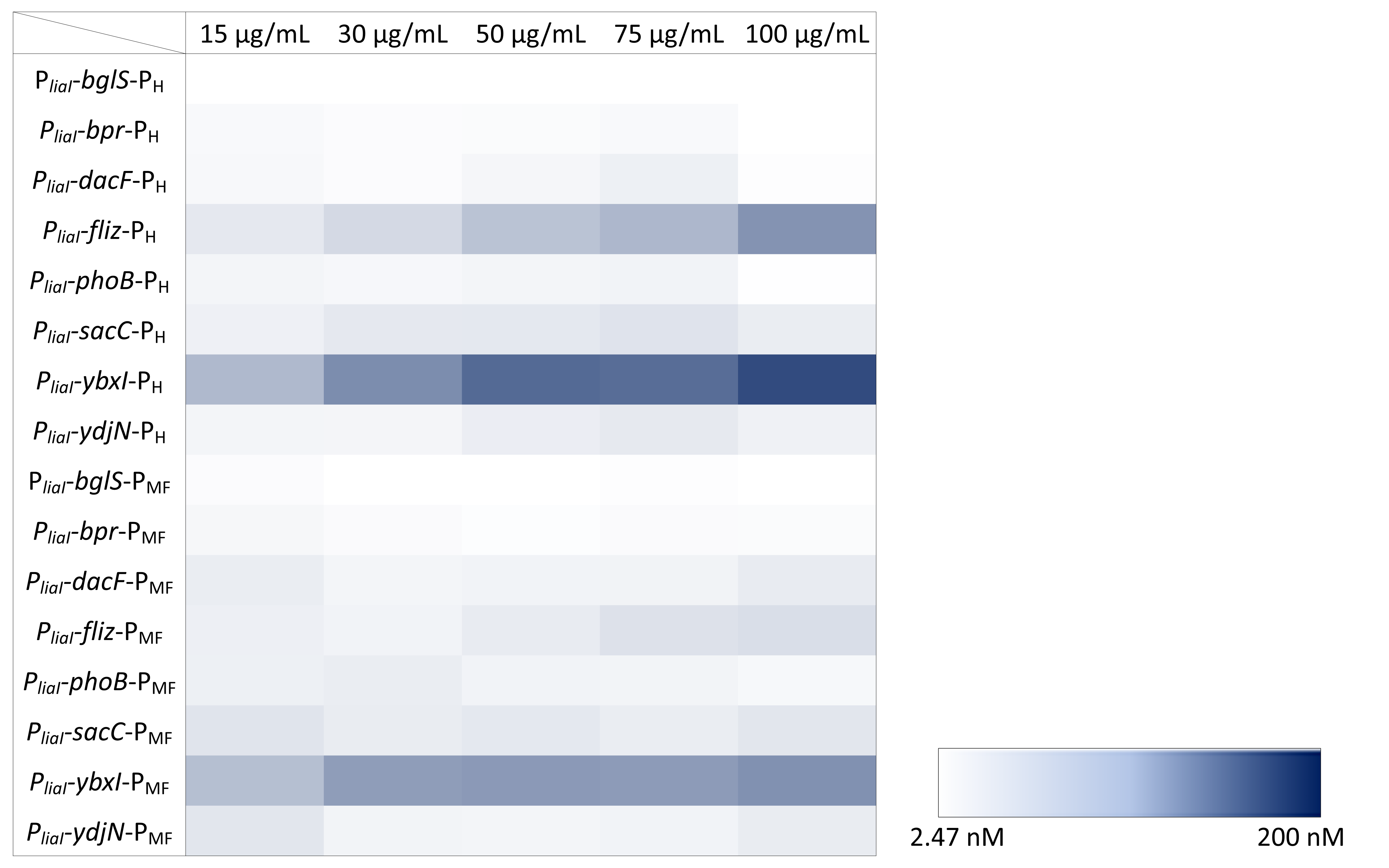


**Figure S4. Identification of best performing signal peptide for P-pheromone production in strains induced by bacitracin.** P-pheromone was produced by inducing the *B. subtilis* producer strains with antibiotic bacitracin (concentration range 15 µg mL^-1^ till 100 µg mL^-1^). Difference in *B. subtilis* strains is in signal peptide and P-pheromone coding sequence identified in screening of signal peptide toolbox as possible best performers. Colour gradient is in the range from minimum standard concentration 2.47 nM to maximum standard concentration of 200 nM P-pheromone.


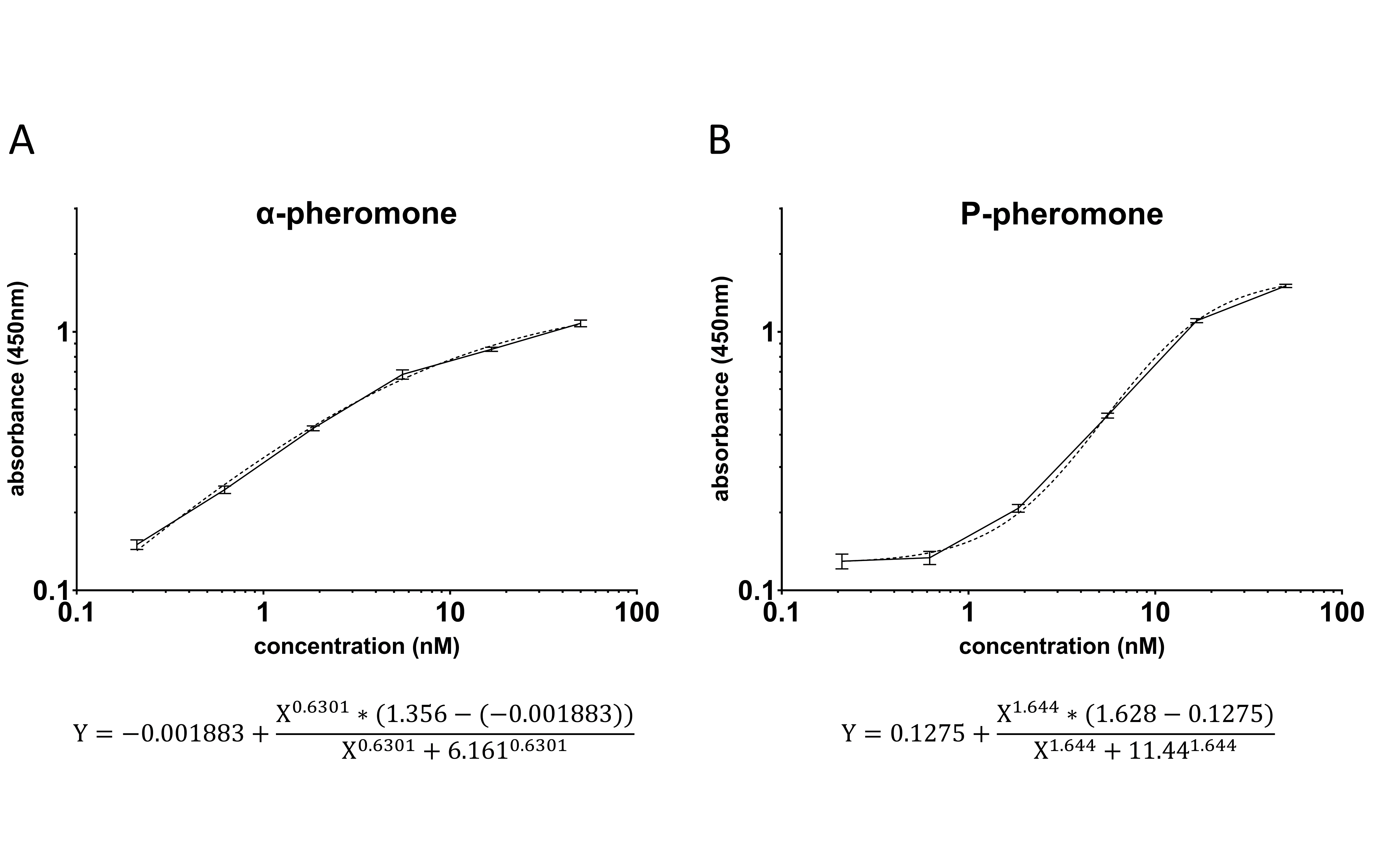


**Figure S5. Four-parameter-dose-response calibration curve of Indirect ELISA assay to determine α- and P-pheromone concentration**. Four-parameter-dose-response nonlinear regression was used to develop calibration curve (dotted line) with appropriate equation to be used for concertation calculation of **A** α-pheromone and **B** P-pheromone in corelation to the measured absorbance.
